# Clinical predictors of flare and drug-free remission in rheumatoid arthritis: preliminary results from the prospective BIO-FLARE experimental medicine study

**DOI:** 10.1136/bmjopen-2024-092478

**Published:** 2025-04-09

**Authors:** Fiona Rayner, Shaun Hiu, Andrew Melville, Theophile Bigirumurame, Amy Anderson, Bernard Dyke, Sean Kerrigan, Andrew McGucken, Jonathan Prichard, Mohadeseh Shojaei Shahrokhabadi, Catharien M U Hilkens, Christopher D Buckley, Iain B McInnes, Wan-Fai Ng, Carl Goodyear, Dawn Teare, Andrew Filer, Stefan Siebert, Karim Raza, Arthur Pratt, Kenneth F Baker, John Isaacs

**Affiliations:** 1 Translational and Clinical Research Institute, Newcastle University, Newcastle upon Tyne, UK; 2 Musculoskeletal Unit, Newcastle Upon Tyne Hospitals NHS Foundation Trust, Newcastle upon Tyne, UK; 3 Population Health Sciences Institute, Newcastle University, Newcastle upon Tyne, UK; 4 Nuffield Department of Primary Care Health Sciences, University of Oxford, Oxford, UK; 5 Institute of Infection, Immunity and Inflammation, University of Glasgow, Glasgow, UK; 6 Institute of Inflammation and Ageing, NIHR Birmingham Biomedical Research Centre, Birmingham, UK; 7 Kennedy Institute of Rheumatology, University of Oxford, Oxford, UK; 8 NHS Greater Glasgow and Clyde, Glasgow, UK; 9 Sandwell and West Birmingham Hospitals NHS Trust, Birmingham, UK

**Keywords:** Rheumatology, Drug Therapy, IMMUNOLOGY

## Abstract

**Abstract:**

**Objectives:**

Huge advances in rheumatoid arthritis (RA) treatment mean an increasing number of patients now achieve disease remission. However, long-term treatments can carry side effects and associated financial costs. In addition, some patients still experience painful and debilitating disease flares, the mechanisms of which are poorly understood. High rates of flare and a lack of effective prediction tools can limit attempts at treatment withdrawal. The BIOlogical Factors that Limit sustAined Remission in rhEumatoid arthritis (BIO-FLARE) experimental medicine study was designed to study flare and remission immunobiology. Here, we present the clinical outcomes and predictors of drug-free remission and flare, and develop a prediction model to estimate flare risk.

**Design, setting and participants:**

BIO-FLARE was a multicentre, prospective, single-arm, open-label experimental medicine study conducted across seven National Health Service Trusts in the UK. Participants had established RA in clinical remission (disease activity score in 28 joints with C reactive protein (DAS28-CRP)<2.4) and were receiving methotrexate, sulfasalazine or hydroxychloroquine (monotherapy or combination).

**Interventions:**

The intervention was disease-modifying anti-rheumatic drug cessation, followed by observation for 24 weeks or until flare, with clinical and immune monitoring.

**Outcome measures:**

The primary outcome measure was the proportion of participants experiencing a confirmed flare, defined as DAS28-CRP≥3.2 or DAS28-CRP≥2.4 twice within 2 weeks, and time to flare. Exploratory predictive modelling was also performed using multivariable Cox regression to understand risk factors for flare.

**Results:**

121 participants were recruited between September 2018 and December 2020. Flare rate by week 24 was 52.3% (95% CI 43.0 to 61.7), with a median (IQR) time to flare of 63 (41–96) days. Female sex, baseline methotrexate use, anti-citrullinated peptide antibody level and rheumatoid factor level were associated with flare. An exploratory prediction model incorporating these variables allowed estimation of flare risk, with acceptable classification (C index 0.709) and good calibration performance.

**Conclusion:**

The rate of flare was approximately 50%. Several baseline clinical parameters were associated with flare. The BIO-FLARE study design provides a robust experimental medicine model for studying flare and remission immunobiology.

**Trial registration number:**

ISRCTN registry 16371380

**STRENGTHS AND LIMITATIONS OF THIS STUDY**

- Rheumatoid arthritis (RA) flare immunobiology is poorly understood. The BIOlogical Factors that Limit sustAined Remission in rhEumatoid arthritis study represents a robust experimental medicine model for the investigation of flare and remission immunobiology in RA.
- We have used routine baseline clinical parameters to develop an exploratory model for the prediction of flare following immunomodulatory drug cessation.
- Limitations include the open-label approach, which could allow for disease flares caused by the nocebo effect.
- A short follow-up time of 6 months means flares after this time were not recorded.
- The small sample size of 121 participants may limit generalisability, although it is comparable with other published literature.

## Introduction

 Rheumatoid arthritis (RA) is a chronic disease characterised by relapsing-remitting episodes of immune-mediated inflammation known as flares, which pose far-ranging negative consequences for patients.^1^ RA flares have been associated with impaired physical function, increased fatigue and reduced quality of life,^2^ as well as serious long-term sequelae, including incremental joint damage^3^ and increased risk of cardiovascular events.^4^ Despite their importance, RA flares remain poorly understood at a mechanistic level and are challenging to investigate scientifically because of their sporadic and unpredictable nature.

Historically most patients with RA suffered from frequent flares, though early diagnosis and rapid initiation of modern regimens of disease-modifying anti-rheumatic drugs (DMARDs) now mean that sustained remission is increasingly an achievable goal in around half of patients. Nevertheless, DMARDs carry risks of drug toxicity, are expensive to prescribe and monitor, and require regular blood testing. International guidelines now advocate consideration of DMARD dose reduction for patients in sustained remission,^5^ although with a risk of arthritis flare in around half of patients who attempt this.^6^,^8^ DMARD cessation provides an experimental human model, acceptable to patients, by which to study the immunobiology of RA flare. In turn, this could identify hitherto elusive biomarkers to guide individualised therapeutic decisions.

The BIOlogical Factors that Limit sustAined Remission in rhEumatoid arthritis (BIO-FLARE) study is an experimental medicine study in which patients with established RA in remission underwent complete DMARD cessation, with the overarching aim of advancing understanding of the biological factors underpinning RA remission and flare through multiparameter immune monitoring.^9^ In this preliminary report, we describe the clinical characteristics and outcomes of the BIO-FLARE cohort, and develop and internally validate an exploratory clinical model to predict the risk of flare at the individual patient level. This model, based on clinical predictors alone, provides a baseline which we will subsequently strengthen by the addition of immune biomarkers, informed by our laboratory studies.

## Methods

BIO-FLARE was a multicentre, prospective, single-arm, open-label, experimental medicine study of complete DMARD cessation in patients with RA who had achieved remission on conventional synthetic DMARDs (csDMARDs: methotrexate, sulfasalazine and hydroxychloroquine; either as monotherapy or in combination).^9^ All participants who fulfilled eligibility criteria stopped all DMARDs at enrolment without tapering. There was no randomisation or control arm, the comparator groups being those who flared versus those who remained in remission. Participants were followed up for 24 weeks or until confirmed flare, whichever occurred earlier. The primary clinical outcomes were time to flare (in days) following DMARD cessation and occurrence of flare (binary) during the 24-week study period. We adhered to the Transparent Reporting of a multivariable prediction model for Individual Prognosis Or Diagnosis reporting guidelines.^10^

### Recruitment criteria

Inclusion criteria included the following: (1) RA fulfilling the 1987 American College of Rheumatology (ACR) or 2010 ACR/European Alliance of Associations for Rheumatology (EULAR) classification criteria; (2) stable dose csDMARDs, with no dose increase in the previous 6 months; and (3) clinical remission according to disease activity score in 28 joints (DAS28) with C-reactive protein (DAS28-CRP)<2.4.^11^ Exclusion criteria included current use of csDMARDs other than methotrexate, sulfasalazine or hydroxychloroquine; use of leflunomide within the previous 12 months (owing to its extended half-life due to enterohepatic recirculation); use of any biological or targeted synthetic DMARDs in the previous 6 months; use of glucocorticoids in the previous 3 months (other than inhaled or topical forms) and any previous ever use of cell-depleting therapies (eg, rituximab). Potential participants were identified by their usual rheumatology teams across seven participating National Health Service (NHS) Trusts in the UK, between September 2018 and December 2020.

### Procedures and definitions

**Figure 1:**
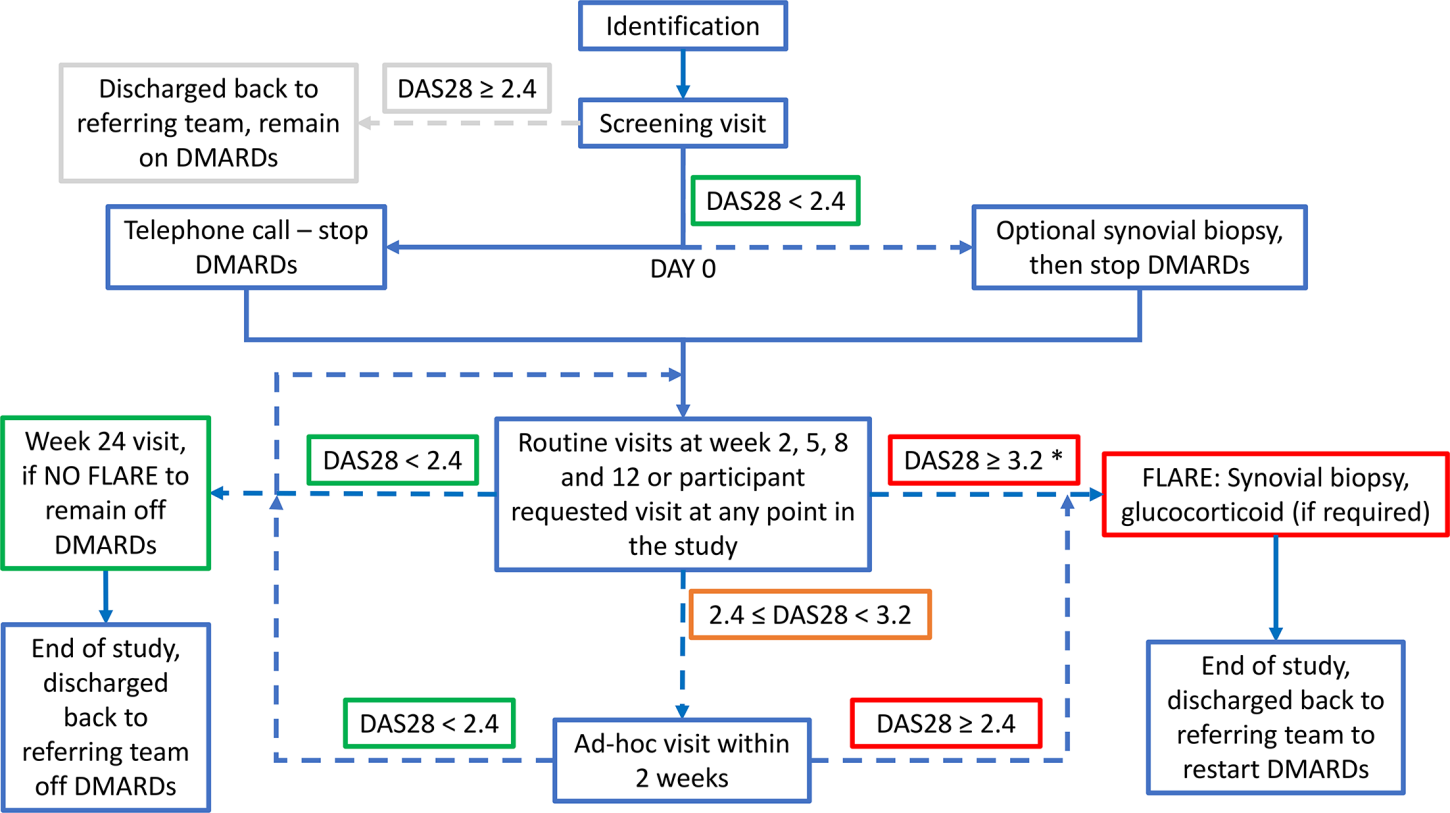
Participant pathway through the study. *Flare defined as DAS28≥3.2 or flare based on clinical discretion. DAS28, disease activity score in 28 joints; DMARD, disease-modifying anti-rheumatic drug.

As shown in figure 1, participants underwent a screening visit to confirm eligibility. Consenting participants stopped all DMARDs immediately once they were deemed eligible, with no dose tapering. An optional baseline ultrasound-guided synovial biopsy was performed in consenting participants prior to DMARD cessation (within 14 days of screening visit). Ultrasound findings did not influence study eligibility. Subsequent study visits took place at weeks 2, 5, 8, 12 and 24 following DMARD cessation. Participant-initiated ad hoc study visits could also be arranged at any time in response to suspected flare. At all study visits, participants underwent clinical assessment, including DAS28-CRP, adverse event (AE) and serious adverse event (SAE) recording, and blood and urine sampling.

Flare was defined as the occurrence of any of the following: (1) DAS28-CRP≥3.2 at any study visit; (2) DAS28-CRP≥2.4 on two occasions within a 14-day period: if DAS28-CRP was≥2.4 but <3.2 at any study visit, then another visit was arranged within 2 weeks, with flare confirmed if DAS28-CRP was≥2.4 at second review; or (3) clinical indication for glucocorticoid rescue therapy and/or DMARD restart despite DAS28-CRP<2.4, for example, for disease activity not captured by DAS28-CRP such as ankle or foot joint synovitis. Clinician discretion was permitted where DAS28-CRP≥3.2 was felt to be driven by identifiable non-RA factors, for example, concurrent infection. In such cases, an ad hoc visit was arranged within 2 weeks, and participants were considered to have remained in remission if subsequent DAS28-CRP was<2.4.

In the event of a confirmed flare, an ultrasound-guided synovial biopsy was performed within 7 days (if there was a joint deemed suitable for biopsy). Systemic or intra-articular glucocorticoid therapy could be administered immediately after biopsy, where indicated. Participants were then referred back to their usual rheumatology team for reinitiation of DMARDs.

### Baseline data collection

Baseline data collected at the screening visit included participant demographics, RA history, current and previous treatments, medical history, including significant comorbidities (Charlson comorbidity index), and patient-reported outcome measures, including functional status (Health Assessment Questionnaire Disability Index) (table 1). A full schedule of events is included in online supplemental table 1.

**Table 1:** Baseline characteristics

		Total study population (n=121)	Modified per-protocol population (n=111)*		Missing data (n=121),N %
			Flare (n=58)	Remission up to week 24 (n=53)	
Age, years	Mean (SD)	64.07 (11.9)	64.76 (11.6)	64.68 (11.3)	0 (0.0)
Sex, female	N (%)	73 (60.3)	41 (70.7)	27 (50.9)	0 (0.0)
Body mass index, kg/m^2^	Mean (SD)	28.20 (5.7)	27.24 (5.3)	29.36 (5.9)	5 (4.1)
Charlson comorbidity index	Median (IQR)	2.00 (1.0, 3.0)	2.00 (1.0, 4.0)	3.00 (1.0, 3.0)	0 (0.0)
Tobacco smoking status	N (%)				0 (0.0)
Current		8 (6.6)	5 (8.6)	3 (5.7)	
Ex-smoker		64 (52.9)	32 (55.2)	26 (49.1)	
Never smoked		49 (40.5)	21 (36.2)	24 (45.3)	
Current alcohol use	N (%)	73 (60.8)	32 (55.2)	36 (67.9)	1 (0.8)
Ethnicity	N (%)				0 (0.0)
White British/Other White		113 (93.4)	55 (94.8)	51 (96.2)	
Asian/Asian British		6 (5.0)	3 (5.2)	1 (1.9)	
Black/Black British- Caribbean		2 (1.7)	0 (0.0)	1 (1.9)	
Highest educational qualification	N (%)				2 (1.7)
GCSEs or equivalent		33 (27.3)	12 (21.0)	18 (34.0)	
A-Level or equivalent		13 (10.7)	7 (12.1)	6 (11.3)	
Undergraduate		20 (16.5)	12 (20.7)	7 (13.2)	
Postgraduate		14 (11.6)	6 (10.3)	5 (9.4)	
NVQ or equivalent		14 (11.6)	5 (8.6)	6 (11.3)	
None of the above		24 (19.8)	15 (25.9)	9 (17.0)	
Not stated or missing		3 (2.5)	1 (1.7)	2 (3.8)	
Employment status	N (%)				0 (0.0)
Full time		30 (24.8)	13 (22.4)	15 (28.3)	
Part time		13 (10.7)	7 (12.1)	4 (7.6)	
Unemployed		3 (2.5)	0 (0.0)	0 (0.0)	
Self-employed		2 (1.7)	1 (1.7)	1 (1.9)	
Retired		71 (58.7)	37 (63.8)	31 (58.5)	
Other		2 (1.7)	0 (0.0)	2 (3.8)	
Time from symptom onset to first DMARD, years	Median (IQR)	0.51 (0.3, 1.3)	0.54 (0.3, 2.1)	0.51 (0.3, 1.0)	4 (3.3)
Time from symptom onset to baseline, years	Median (IQR)	6.33 (4.5, 12.4)	6.34 (5.0, 13.7)	6.17 (3.9, 10.8)	4 (3.3)
Time from RA diagnosis to baseline, years	Median (IQR)	5.48 (3.7, 10.5)	5.48 (4.2, 10.7)	5.36 (3.3, 9.7)	2 (1.7)
MTX use at baseline	N (%)	101 (83.5)	53 (91.4)	39 (73.6)	0 (0.0)
MTX dose, mg/week	Median (IQR)	15 (12.5, 20)	15 (12.5, 20)	15 (12.5, 20)	0 (0.0)
MTX monotherapy	N (%)	72 (59.5)	37 (63.8)	28 (52.8)	0 (0.0)
MTZ+SZN	N (%)	5 (4.1)	3 (5.2)	2 (3.8)	0 (0.0)
MTX+HCQ	N (%)	22 (18.2)	11 (19.0)	9 (17.0)	0 (0.0)
MTX+SZN + HCQ	N (%)	2 (1.7)	2 (3.5)	0 (0.0)	0 (0.0)
SZN monotherapy	N (%)	10 (8.3)	3 (5.2)	7 (13.2)	0 (0.0)
HCQ monotherapy	N (%)	8 (6.6)	2 (3.5)	5 (9.4)	0 (0.0)
SZN+HCQ	N (%)	2 (1.7)	0 (0.0)	2 (3.8)	0 (0.0)
Previous biological therapy	N (%)	1 (0.8)	1 (1.7)	0 (0.0)	0 (0.0)
Corticosteroid use in the past 12 months	N (%)	7 (5.8)	3 (5.2)	4 (7.6)	
Any		7 (5.8)	3 (5.2)	4 (7.6)	0 (0.0)
Oral		3 (2.5)	1 (1.7)	2 (3.8)	0 (0.0)
Intramuscular		1 (0.8)	0 (0.0)	1 (1.9)	0 (0.0)
Intra-articular		2 (1.7)	2 (3.5)	1 (1.9)	0 (0.0)
RF positive	N (%)	67 (56.3)	42 (72.4)	19 (36.5)	2 (1.7)
ACPA positive	N (%)	76 (66.7)	45 (77.6)	25 (49.0)	7 (5.8)
RF, IU/mL	Median (IQR)	32.00 (0.0, 94.1)	53.15 (14.0, 130.0)	12.65 (0.0, 40.1)	2 (1.7)
ACPA, U/mL	Median (IQR)	96.50 (1.1, 300.0)	207.00 (31.0, 306.5)	1.70 (0.8, 196.0)	7 (5.8)
DAS28-CRP	Mean (SD)	1.61 (0.3)	1.62 (0.3)	1.60 (0.3)	0 (0.0)
ACR/EULAR 2011 Boolean remission	N (%)	74 (61.2)	38 (65.5)	31 (58.5)	0 (0.0)
ACR/EULAR Boolean 2.0 remission	N (%)	95 (78.5)	48 (82.8)	41 (77.4)	0 (0.0)
SDAI remission	N (%)	101 (84.9)	49 (86.0)	45 (84.9)	2 (1.7)
HAQ-DI	Median (IQR)	0.00 (0.0, 0.6)	0.13 (0.0, 0.7)	0.00 (0.0, 0.4)	0 (0.0)
Follow-up time, days	Median (IQR)	115.5 (55.5, 167.5)	63.0 (41.0, 96.0)	168.0 (167.0, 174.0)	0 (0.0)

* The modified per-protocol population includes all participants with known outcome status and excludes those lost to follow-up (n=7) or withdrawn (n=3) before week 24 visit.

ACPA, anti-citrullinated peptide antibody; ACR, American College of Rheumatology; DAS28-CRP, disease activity score in 28 joints with C reactive protein; DMARD, disease-modifying anti-rheumatic drug; EULAR, European Alliance of Associations for Rheumatology; GCSE, General Certificate of Secondary Education; HAQ-DI, Health Assessment Questionnaire Disability Index; HCQ, hydroxychloroquine; MTX, methotrexate; NVQ, National Vocational Qualification; RA, rheumatoid arthritis; RF, rheumatoid factor; SDAI, simplified disease activity index; SZN, sulfasalazine.

### Statistical analysis

The primary outcome for the current study was time to disease flare (in days). The Kaplan-Meier estimate of the survivor curve was computed along with numbers at risk at the scheduled visit dates (weeks 2, 5, 8, 12 and 24). Participants who were lost to follow-up or withdrawn from the study were censored at the last available visit.

16 candidate baseline variables were considered for exploratory prediction model inclusion: age, sex, disease duration, time from symptom onset to first DMARD, baseline methotrexate use, glucocorticoids within 3–12 months of baseline visit, baseline rheumatoid factor (RF) level, baseline anti-citrullinated peptide antibody (ACPA) level, DAS28-CRP, ACR/EULAR Boolean remission status,^12^ education level, employment status, body mass index, smoking status, alcohol intake and Charlson comorbidity index. These were chosen based on prior knowledge and before reviewing study data. Owing to the presence of some missing data points, analyses were performed with 10 imputed data sets using multiple imputation by chained equations.^13^

To provide clinical context, univariate analyses were performed to assess the strength of association between each candidate variable and time to flare, with HRs and 95% CIs determined.

A predictive model for flare containing baseline clinical variables was built using a Cox proportional hazards model following a sequential process of variable selection, estimation of shrinkage and internal validation, described in detail in online supplemental materials. Predictive performance was internally validated using bootstrapping and evaluated with optimism-corrected indices of discrimination (*C* index) and calibration.^14^,^16^ We report our predictive model as an equation for calculating the prognostic index (PI), representing an individual’s ‘propensity’ to flare, and a baseline survival function, which together allow calculation of estimated risk of flare by a given time following DMARD cessation.

### COVID-19 mitigation and sensitivity analysis

The latter stages of the BIO-FLARE study overlapped with the onset of the COVID-19 pandemic, meaning some follow-up visits were disrupted. A mitigation strategy was adopted whereby affected participants received telephone consultations when their study visits were due, with assessments of flare/remission status based on participant-reported symptoms rather than DAS28-CRP, and face-to-face visits reserved for those with suspected flare. Seven participants were lost to follow-up during this period, while four had telephone consultations up to week 24. For our primary analyses, participants with telephone consultations up to week 24, and no symptoms of flare, were classified as having remained in remission. A sensitivity analysis of our predictive modelling process was conducted using last face-to-face study visits only (ie, last available DAS28-CRP).

### Study subpopulations

Overall baseline characteristics and AE data are described for all participants who stopped DMARDs (n=121, the total study population). Time-to-event analyses, including predictive modelling, were performed for participants with ≥1 follow-up visit (n=120, the analysis population which excludes one participant who withdrew soon after baseline because of the COVID-19 pandemic). Flare rate was calculated for participants with confirmed flare or remission status (n=111, which we term the ‘modified per-protocol’ population following the COVID-19 mitigation strategy), that is, excluding 10 participants who did not experience flare but withdrew (n=3) or were lost to follow-up (n=7) before week 24.

### Patient and public involvement

The Newcastle Patient and public Involvement in Musculoskeletal reSearch group was consulted at the planning stage of the project. The importance of the research topic and design of the study protocol was informed by their views and discussions. Clinical results from the study have been presented at national versus arthritis meetings with patient partners present. We will present more results of the study to local, regional and national PPIE groups as they become available.

## Results

### Baseline characteristics and adverse events

**Figure 2:**
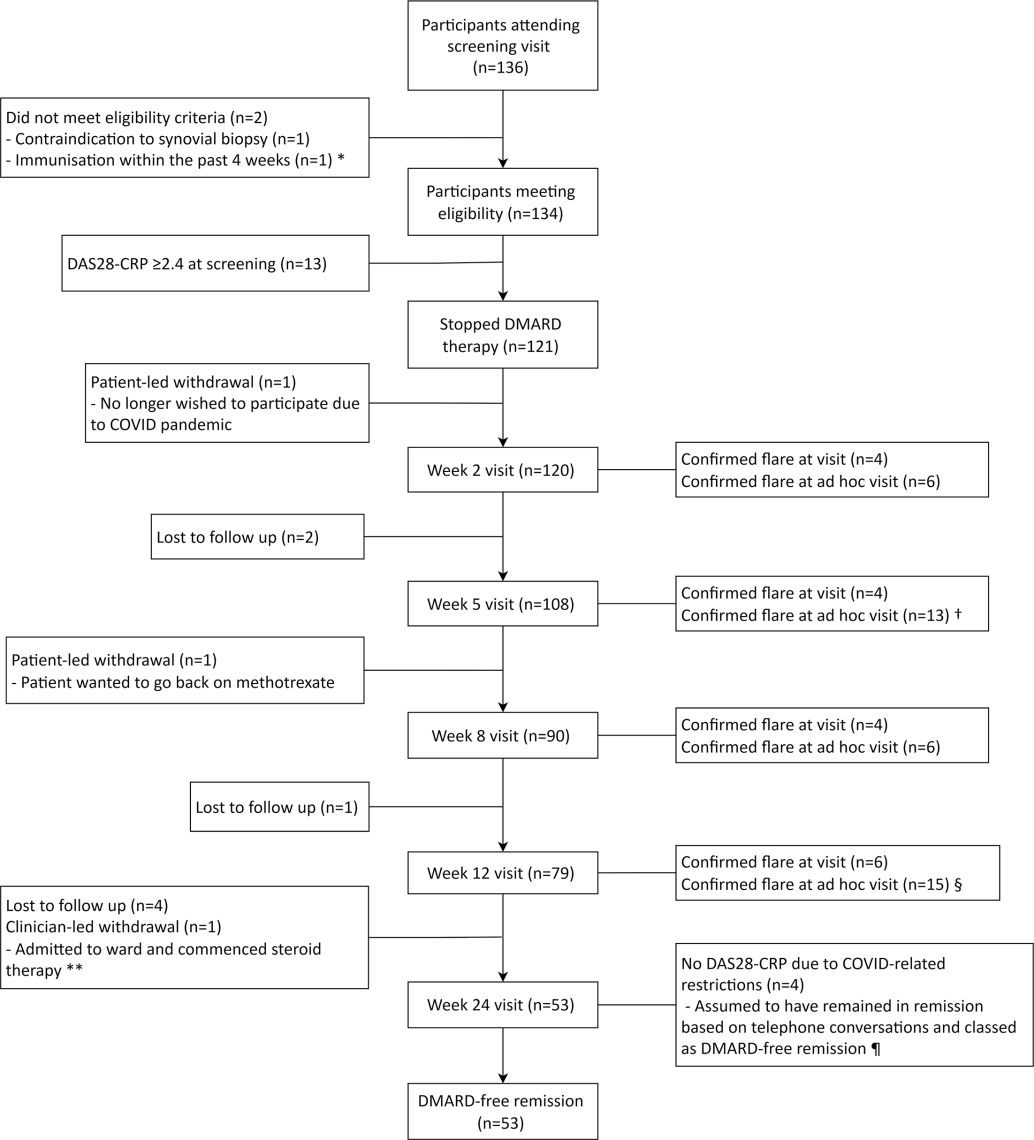
Participant flow diagram. * Participant discovered to have had an immunisation prior to screening at their week 2 visit; † n=2 participants flared based on clinical discretion at face-to-face visit; § n=1 flare based on clinical discretion at face-to-face visit; ** participant was censored at day 84 visit as discovered to have an intercurrent illness at week 24 visit and was withdrawn from the study; ¶ participants had last face-to-face visits at week 2 (n=1), week 5 (n=1), ad hoc visit after week 5 (n=1) and week 12 (n=1) visits. DAS28, disease activity score in 28 joints; DMARD, disease-modifying anti-rheumatic drug.

121 participants met the inclusion criteria, including DAS28-CRP<2.4, and stopped DMARD therapy (figure 2). The overall baseline characteristics are presented in table 1, along with the baseline characteristics for participants who flared and those who remained in remission at 24 weeks (n=111, the modified per-protocol population). For the total study population, mean (SD) age was 64.1 (11.9) years, 60.3% were female and median (IQR) disease duration was 6.3 (4.5–12.3) years. 67/119 (56.3%) were RF-positive and 76/114 (66.7%) were ACPA-positive, with 64/113 (56.6%) double-positive. Only one participant had previous biological therapy (etanercept, stopped 7.5 years before study entry). 101/121 participants (83.5%) were treated with methotrexate at baseline (monotherapy or combination use) with a median (IQR) dose of 15 (12.5–20) mg weekly. Of 20 participants not on methotrexate at baseline, 7/20 had previously received methotrexate treatment. Mean (SD) baseline DAS28-CRP was 1.61 (0.32); 61.2%, 78.5% and 84.9% fulfilled ACR/EULAR Boolean remission criteria, Boolean 2.0 remission criteria^17^ and simplified disease activity index (SDAI) remission criteria at baseline, respectively. There were 155 AEs (online supplemental table 2), 4 SAEs relating to hospitalisations (online supplemental table 3) and no deaths. The four SAEs were all considered to be unrelated to study participation or procedures.

### Flare characteristics

**Figure 3:**
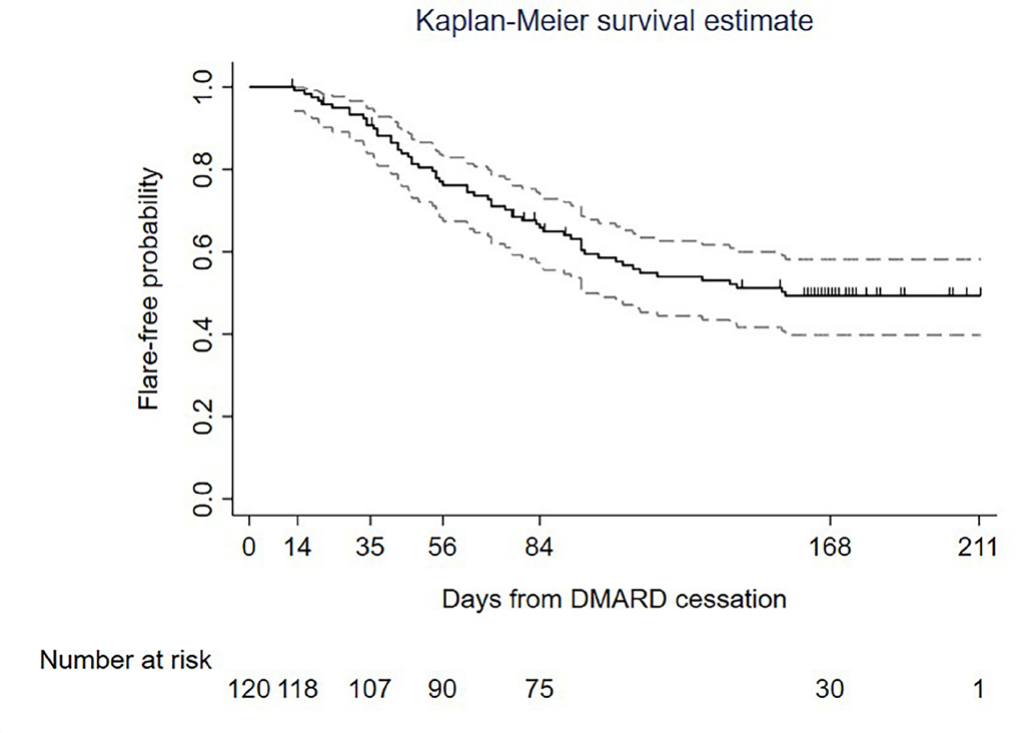
Kaplan-Meier plot of flare-free probability in the analysis cohort. Solid black line is the Kaplan-Meier estimate of the flare-free function, the grey dashed lines are the 95% CI and black vertical marks indicate censoring. Outcomes defined as per primary analyses. A Kaplan-Meier plot including only data from final face-to-face study visits (sensitivity analysis) is included as online supplemental figure 2. DMARD, disease-modifying anti-rheumatic drug.

The flare rate at 24 weeks (168 days) was 52.3% (58/111, 95% CI 43.0 to 61.7). Flare-free probability is presented in a Kaplan-Meier plot in figure 3 (Kaplan-Meier plot of flare-free probability based on face-to-face visits is available in online supplemental figure 2). For the 58 participants who experienced flare, median time to flare was 63 days (IQR 41–96 days, range 13–155 days).

Mean (SD) DAS28-CRP at time of flare was 3.81 (0.78). DAS28-CRP components at time of flare were as follows: median (IQR) tender joint count 4 (1–5), swollen joint count 2 (1–3), CRP 8.0 (4.5–14.4) mg/dL; mean (SD) patient global health Visual Analogue Score (VAS) 48.4/100 (22.7).

Confirmation of flare was based on a single DAS28-CRP result≥3.2 in 39/58 cases (of which 18 were scheduled study visits and 21 were ad hoc visits), two DAS28-CRP results≥2.4 within a 14-day period in 16/58 cases (of which 8 had DAS28-CRP≥3.2 at the second visit) and clinician discretion in 3/58 cases (described in online supplemental table 4).

### Univariate Cox proportional hazards models

Of the 16 variables considered in univariate analyses, female sex, methotrexate use at baseline, RF level, ACPA level and longer time from symptom onset to first DMARD were statistically significantly associated with time to flare (table 2).

**Table 2:** Univariate analysis of candidate baseline variables predicting flare in the analysis cohort

		Complete case analysis			Multiple imputation with chained equations (n=120)	
		Available n	HR (95% CI)	P value	HR (95% CI)	P value
	Reference					
Age		120	1.00 (0.98 to 1.02)	0.99		
Male sex	Female	120	0.54 (0.31 to 0.95)	0.03		
Years from diagnosis to baseline visit		118	1.01 (0.99 to 1.04)	0.37	1.01 (0.98 to 1.04)	0.47
Years from symptom onset to first DMARD		112	1.04 (1.00 to 1.07)	0.04	1.03 (1.00 to 1.07)	0.06
Methotrexate use at baseline	No	120	2.92 (1.16 to 7.31)	0.02		
RF level at baseline, per 10 IU/mL		118	1.03 (1.01 to 1.06)	0.001	1.03 (1.01 to 1.06)	0.001
ACPA level at baseline, per 10 U/mL		113	1.03 (1.01 to 1.04)	0.01	1.03 (1.01 to 1.04)	0.01
DAS28-CRP		120	1.14 (0.51 to 2.52)	0.75		
ACR/EULAR 2011 Boolean remission at baseline	Not in remission	120	1.16 (0.68 to 2.00)	0.59		
A-level and above education	GCSE and under	117	1.15 (0.68 to 1.94)	0.59	1.14 (0.67 to 1.94)	0.62
BMI		115	0.95 (0.90 to 1.01)	0.08	0.96 (0.90 to 1.01)	0.11
Current smoker	Never or ex-smoker	120	1.04 (0.41 to 2.60)	0.94		
Current alcohol use	No	119	0.63 (0.37 to 1.05)	0.07	0.63 (0.38 to 1.06)	0.09
Charlson comorbidity index		120	1.04 (0.88 to 1.22)	0.66		
Glucocorticoid use in the past 12 months from baseline	No	120	0.78 (0.24 to 2.48)	0.67		

Employment variable was not included in the imputation model due to convergence issues due to low frequency in the unemployed subgroup. Variables with no missing data have empty rows under the multiple imputation with chained equations column because estimates will be identical to the complete case analysis. HRs for continuous variables are calculated per one unit increase unless otherwise stated.

ACPA, anti-citrullinated peptide antibody; ACR, American College of Rheumatology; BMI, body mass index; DAS28-CRP, disease activity score in 28 joints with C reactive protein; DMARD, disease-modifying anti-rheumatic drug; EULAR, European Alliance of Associations for Rheumatology; GCSE, General Certificate of Secondary Education; RF, rheumatoid factor.

### Exploratory prediction model

Our prediction modelling procedure, including variable selection, resulted in the inclusion of sex, methotrexate use at baseline, RF level and ACPA level into the prediction model (see online supplemental table 5). A square root transformation of RF and two non-linear expressions of ACPA (inverse of ACPA and inverse square root of ACPA) were chosen as the best-fitting transformations. Thus, our prediction model consisted of five terms: sex, methotrexate use, (RF+0.1)^0.5^, (ACPA+0.1)^-1^ and (ACPA+0.1)^-0.5^.

The predicted probability of a flare within *t* days after DMARD cessation can be computed as


Predicted risk of flare by t days after DMARD cessation=1− S^0(t)e^xp(PI)

where ${\hat{S}}_{0}\left(t\right)$ is the estimated baseline survival function at time *t*, PI is the prognostic index and *exp*(.) is the exponential function. The value of ${\hat{S}}_{0}\left(t\right)$ at *t*=168 days after DMARD cessation is 0.672. Additional values at 30, 60, 90 and 120 days are available in online supplemental materials (‘Statistical analysis’ section). The PI is computed as


$$\begin{array}{ll} PI\;= & \;\left(-0.55814869\times \;Sex\;\right)+\left(1.05775338\times \;Methotrexate\;use\right)+\\ \left(0.03734463\times \sqrt{RF+0.1}\right)+\;f\left(ACPA\right) \end{array}$$

where $f\left(ACPA\right)=\;\left(\frac{0.55920681}{ACPA+0.1}\right)-\left(\frac{1.86737912}{\sqrt{ACPA+0.1}}\right)$ sex coded as female=0, male=1, methotrexate use coded as no=0, yes=1.

Thus, as an example, for a female patient, who was not taking methotrexate at baseline, has an RF measurement of 60 IU/mL and an ACPA measurement of 150 IU/mL, the PI would be 0.141, and the predicted risk of flare by 168 days after DMARD cessation would be 36.7%.

The model had an optimism-corrected C index of 0.709 (95% CI 0.647 to 0.771) and calibration slope of 1.00 (95% CI 0.495 to 1.506), indicating acceptable classification performance and good agreement between estimates of flare risk and observed risk (see ‘Statistical analysis section’ of online supplemental materials and online supplemental figure 1). The sensitivity analysis of the prediction model, using last face-to-face study visits, demonstrated comparable predictive properties (C-index 0.707 (95% CI 0.643 to 0.771), calibration slope 0.996 (95% CI 0.494 to 1.497)).

## Discussion

BIO-FLARE is an experimental medicine study designed to provide insights into the biological processes that trigger episodes of flare in patients with RA. The ability to compare patients who remain in remission on DMARD cessation with those who flare provides a well-controlled biological model. In this current work, we describe the clinical characteristics of the BIO-FLARE cohort, report the main clinical outcomes and explore predictors of flare among routine baseline clinical parameters.

Approximately 50% of participants experienced a flare over the 6-month study period, which is similar to the results of previous csDMARD withdrawal studies in RA.^618^,^20^

Among baseline parameters, we identified methotrexate use, female sex, RF level and ACPA level as significant predictors of flare following DMARD cessation. Higher RF and ACPA levels have been associated with adverse outcomes in RA, including radiographic progression, and may indicate a more aggressive disease phenotype,^21^ and seropositivity is associated with progression from pre-clinical to clinically apparent RA,^22 23^ which might be analogous mechanistically to flare. Similarly, female sex has been associated with progression to RA from early undifferentiated arthritis.^24^ The increased risk of flare following methotrexate cessation might reflect more severe underlying disease, confounding by indication for other reasons (ie, reasons for avoiding or previous discontinuation of methotrexate might be protective), and/or a particular pharmacodynamic mechanism of action that leads to highly effective suppression of disease activity but not true biological remission. Longer time from symptom onset to DMARD initiation had a borderline association with flare and was not selected for inclusion in the final model, but does hint at early and effective treatment modifying the probability of achieving drug-free remission, in line with the ‘window of opportunity’ concept.^25 26^

The association between female sex, RF and ACPA positivity and flare has been noted in previous DMARD withdrawal studies and lends face validity to our results.^18 27^ In the BioRRA study, a precursor to BIO-FLARE, RF positivity and longer time from diagnosis to first DMARD were also associated with flare, while a borderline association was seen for baseline methotrexate use.^6^

Unlike previous DMARD withdrawal studies, we adopted a predictive modelling approach towards our baseline clinical parameters and developed and internally validated an exploratory prediction model that allows estimation of risk of flare for an individual patient by a given time following csDMARD cessation. To our knowledge, no comparable clinical model has been described previously in this context. Our final model had acceptable performance in classifying flare versus remission, with good agreement overall between observed and predicted risks. An easy-to-use online version of the formula can be found at https://research.ncl.ac.uk/bioflare/outputs/. Using this tool, sex, methotrexate use, baseline RF and ACPA values can be entered and a predicted risk of flare at 90 or 168 days obtained. Given the lack of external validation, we do not recommend that this tool is used to guide clinical decisions. Nevertheless, in producing a predictive model using only routinely collected data, we present a benchmark against which future molecular or multimodal models can be compared.

The strengths of our study include the number of participants, which compares favourably with previous DMARD withdrawal studies, the prospective study design and the minimal missing data among baseline parameters. Our predictive modelling followed a robust statistical approach, thereby reducing the risk of bias from sensitivity to sampling variability through bootstrapping and overfitting through shrinkage. Nevertheless, our study does have some limitations. BIO-FLARE included participants on csDMARDs only, meaning the relevance of our findings to patients treated with biological or targeted synthetic DMARDs is uncertain. However, recent studies suggest that up to 40–50% of real-world patients with RA are treated with csDMARDs alone,^28 29^ and it is possible that the immunobiological mechanisms underlying flare may be intrinsic to RA disease processes and thus independent of DMARD treatment. The DAS28-CRP score, used in our study to define remission and flare, has been criticised in the past for being overly permissive of active inflammation.^30^ However, we used a stringent cut-off of <2.4 and found similar percentages of participants achieving Boolean and SDAI remission at baseline between subsequent flare and remission groups, suggesting flare was not simply driven by discrepancies in uncaptured initial disease activity. The open-label treatment withdrawal creates a risk of flares driven by the nocebo effect, but this was a pragmatic study design that reflects clinical practice. Musculoskeletal imaging was not performed at baseline, meaning the predictive potential of radiographic erosions, or ultrasonographic synovitis/tenosynovitis could not be assessed. The COVID-19 pandemic limited face-to-face assessments for a relatively small proportion of study participants, but the close similarity between our primary and sensitivity analyses suggests our mitigation strategy was valid, without an obvious impact on the performance of the prediction model. Finally, the 6-month follow-up period means that longer-term outcomes, such as the occurrence of flares beyond 24 weeks, response to csDMARD reinitiation and long-term sequelae that might be associated with flares, were not captured by the current study. There was also no dedicated long-term follow-up to identify any participants who did not quickly regain remission following a flare in the study. This, along with longer-term follow-up of those who exited the study in remission, would be interesting further work to explore. Other published work has shown that remission is quickly regained in the majority of participants who experience mild disease flares when tapering or stopping DMARDs.^31^

In conclusion, approximately half of the patients with RA in remission on csDMARDs experienced a flare within 6 months of stopping therapy, with a median time-to-flare of 9 weeks. Among baseline clinical parameters, RF and ACPA levels, female sex and methotrexate use were found to be predictive of flare. Our predictive model allows estimation of risk of flare at the individual level based on clinical parameters alone. We will subsequently strengthen this by the addition of immune biomarkers emerging from our BIO-FLARE laboratory analyses.

## Supplementary material

10.1136/bmjopen-2024-092478 online supplemental file 1

## Data Availability

Data are available upon reasonable request. All data relevant to the study are included in the article or uploaded as supplementary information.
