# Supplementary material for: Clinical predictors of flare and drug-free remission in rheumatoid arthritis: preliminary results from the prospective BIO-FLARE experimental medicine study
Source: BMJ Open. 2025 Apr 9;15(4):e092478. doi: 10.1136/bmjopen-2024-092478 (PMC11987156; doi:10.1136/bmjopen-2024-092478)
Supplement: online supplemental file 1 [file bmjopen-15-4-s001.docx]

# Clinical predictors of flare and drug-free remission in rheumatoid arthritis: preliminary results from the prospective BIO-FLARE experimental medicine study

# – Supplementary Material

Contents

[Statistical analysis - Predictive model with baseline risk factors 1](#_Toc189825574)

[Supplementary Table 1: Schedule of events in the BIO-FLARE study. 6](#_Toc189825575)

[Supplementary Table 2: All adverse events. 9](#_Toc189825576)

[Supplementary Table 3: Serious adverse events 9](#_Toc189825577)

[Supplementary Table 4: Characteristics of patients adjudged to have flared based on clinician discretion (n=3). 9](#_Toc189825578)

[Supplementary Table 5: Bootstrap inclusion frequencies 10](#_Toc189825579)

[Supplementary Figure 1: Calibration plot at day 168 11](#_Toc189825580)

[Supplementary Figure 2: Kaplan-Meier plot of flare-free survival based on face-to-face visits. 12](#_Toc189825581)

[Supplementary References 13](#_Toc189825582)

## Statistical analysis - Predictive model with baseline risk factors

The primary analysis of this study involved the building and presentation of a prediction model containing baseline risk factors of RA flare through three steps: 1) Variable selection for the prediction model; 2) Assessment of non-linear forms for continuous predictors; 3) Estimation of a shrinkage factor to reduce overfitting; 4) Internal validation of the prediction model; and 5) Presenting the predicted probability of flare as a function of the prognostic index and time. For ease of reporting, we denote lower case *i* to represent an imputed dataset (*i* = 1, 2, …, *I*) and lower case *j* to denote a bootstrap resample of an imputed dataset (*j* = 1, 2, …, *J*).

***0. Justification of maximum number of predictors in model.*** We computed the maximum number of variables that may be included in our prediction model, under the assumption that there would be minimal overfitting (i.e. an expected shrinkage factor ≥0.90) and a sample size of n=120 (1, 2). We used the lower bound of the 95% confidence interval of the area under curve statistic from a previous baseline prediction model (3) for RA flare as an approximation to the C index, forming the basis for our calculations. The computation indicated an upper limit of nine predictor variables.

We assumed that:

- There would be approximately E = 60 events based on the pre-specified assumption that half of participants will experience a flare (3) and the current sample size of approximately n = 120 (due to the early closure of the study); and
- A global shrinkage factor of SVH = 0.90, indicating that, given the number of events, a model with those $p$ predictors but without shrinkage would only be slightly over-fitted to the data.

Let C $\approx$ 0.91. A value of Royston’s D statistic (Equation 22) was computed as:

$$D=5.50\left( C-0.5 \right)+10.26{(C-0.5)}^{3}=2.962$$

An apparent value of R^2^_D_ (Equation 21) was computed as:

$$R_{D\_app}^{2}= \frac{\frac{\pi}{8}D^{2}}{\frac{\pi^{2}}{6}+ \frac{\pi}{8}D^{2}}=0.679$$

R^2^_D_app_ was then used as a proxy for an apparent value of R^2^_Royston_ to compute an apparent value of R^2^_O’Quigley_ (Equation 20):

$$R_{O'Quigley\_app}^{2}= \frac{-\frac{\pi^{2}}{6}R_{Royston\_app}^{2}}{\left( 1- \frac{\pi^{2}}{6} \right)R_{Royston\_app}^{2}-1}=0.775$$

A likelihood ratio (LR) statistic was then computed (Equation 18) with *E* = 60:

$$LR= -E\times LN\left( 1- R_{O^{'}Quigle{y\_}_{app}}^{2} \right)=89.515$$

The number of predictors *p* was computed with a rearrangement of Equation 3 and *S_VH_* = 0.90:

$$p=\left( 1- S_{VH} \right)\times LR=8.952 \approx9$$

* All figures are rounded to 3 d.p.

***1. Variable selection.***

A subset of 16 baseline variables was first selected as risk factors for flare based on biological plausibility and previous literature.

- Age at baseline
- Sex (Female vs. Male)
- Time from diagnosis to baseline in years
- Time from symptom onset to DMARD commencement in years
- Methotrexate use (Yes vs. No)
- Rheumatoid factor (RF) in IU/ml
- Anti-citrullinated peptide antibody (ACPA) in U/ml
- DAS28-CRP
- ACR/EULAR Boolean Remission (In remission vs. Not in remission) (4)
- Education (GCSE and under vs. A-levels and above (including national vocational qualifications))
- Any employment (Unemployed vs. Employed or retired)*
- Body mass index in kg/m^2^
- Current smoking (Never or ex-smoker vs. Current smoker)
- Any alcohol use (Yes vs. No)
- Charlson comorbidity index (5)
- Corticosteroid use

* Due to the problems with convergence for the employment variable observed in the univariate analyses, this variable was dropped from all multiple imputation and prediction modelling steps.

Owing to the presence of missing data, we performed all analyses with *I*=10 imputed datasets using multiple imputation by chained equations (MICE) (6). Predictive mean matching was used as the imputation method as it has been shown to produce the less biased estimates in and better predictive performance of Cox PH models than complete case analyses or single imputation methods when the missing covariate data rate is >10% (7). The 15 candidate predictors, the outcome indicator, and the Nelson-Aalen estimate of the cumulative baseline hazard were included in the imputation model (8).

For each imputed dataset *i*, *J*=200 datasets were generated via bootstrapping (resampling with replacement). The use of MI followed by bootstrapping allows one to account for uncertainty due to missing data and uncertainty due to sampling variability during variable selection (9). Within each MICE-cum-bootstrap dataset, the 15 variables were included in a penalised Cox proportional hazard model (PH) using elastic net penalty for further variable selection whilst addressing issues of multicollinearity. We used 10-fold cross validation to select an optimal mixing parameter α and tuning parameter λ. We varied α from 0.1 to 0.9 at increments of 0.1. At each value of α, a value of the tuning parameter λ was selected at one standard error from the value of that λ associated with the regularised model with the smallest out-of-fold concordance statistic *C*. From the nine sets of α_k_, λ_k_, and *C*_k_ {*k* = 1, 2,… 9}, the optimal values of α and λ was obtained from searching for the largest *C*_k_. We assessed the stability of the candidate predictors by inspecting their bootstrap inclusion frequencies (BIF) across all 2000 datasets. The idea is that if a baseline risk factor was associated with RA flare, it should consistently, or at least in a large number of times, be selected into the final model even under “perturbations” in the data. Bootstrap resampling has been found to be a useful method of mimicking these modifications for Cox PH models (10-12). The BIF of each candidate variable is the number of times it was included in the regularised model at the optimal values of α and λ across all bootstrap replications of an imputed dataset. We identified stable risk factors as predictors with >60% BIF across the average of all imputations (up to a limit of nine predictors). The value of 60% was decided a priori before analyses were conducted.

**2. Assessment of non-linear forms for continuous covariates.** For each imputed dataset, we conducted univariable fractional polynomials (FP) to explore the best-fitting non-linear functional form of the selected continuous predictors (RF and ACPA). We only explored non-linear functional forms following the variable selection strategy. This was because we were not certain how to setup the elastic net variable selection process in a way such that if one of the non-linear terms for a given continuous variable (e.g., age) had its coefficient shrunk to zero during penalisation, we would also want the other non-linear terms to do the same. Thus to simplify the process, we decided to conduct the variable selection with linear terms only, then apply non-linear transformations on continuous variables that ‘passed’ variable selection.

For each covariate, we explored first-degree and second-degree FPs in a univariate Cox model using the RA2 closed test procedure with a nominal α value of 0.10. To avoid numerical issues, a constant of 0.1 was added to the continuous variables (13). For RF, all imputations suggested a first-degree fractional polynomial with a square root transformation. For ACPA, 4/10 imputations suggested second-degree fractional polynomials with inverse and negative square root transformations respectively, 3/10 suggested a first-degree fractional polynomial with a log-transformation respectively, and 3/10 suggested second-degree fractional polynomials with two terms with inverse transformations respectively. Thus, based on the transformation suggested most frequently across imputations, we decided on the second-degree fractional polynomials with inverse and negative square root transformations.

**3. Estimation of shrinkage factor.** To reduce the effects of overfitting, for each imputation *i* we estimated a shrinkage factor *S_i_* using bootstrap estimation with *J*=200 resamples. A recent study demonstrated that for studies with small sample sizes, bootstrapping may be preferred over the heuristic shrinkage or penalised regression methods to obtain a more reliable estimate of a shrinkage factor for small sample sizes (14). To illustrate the procedure, consider a particular bootstrap resample *j* for a particular imputed dataset *i*. The survival outcome of bootstrap dataset *j* is regressed on the stable risk factors in the bootstrap sample in a Cox regression model, and the coefficients are saved. A linear predictor (LP) is then calculated as the linear combination of the values of the stable risk factors in the imputed dataset *i*, weighted by the coefficients derived from the bootstrap sample earlier. The outcome of the imputed dataset *i* is regressed on the LP and the coefficient of the LP is saved. The value of *Si* is the average of all the coefficients of LP across the *J* bootstraps. The estimated shrinkages factors ranged from 0.831 to 0.891, which were generally close to the assumed 0.90 uniform shrinkage assumed when computing the maximum number of predictors allowable in our prediction model.

| **Imputation** | **Estimated shrinkage factor** |
| --- | --- |
| 1 | 0.857 |
| 2 | 0.835 |
| 3 | 0.842 |
| 4 | 0.841 |
| 5 | 0.891 |
| 6 | 0.843 |
| 7 | 0.831 |
| 8 | 0.845 |
| 9 | 0.831 |
| 10 | 0.858 |
| **Average** | **0.848** |

**4. Internal validation.** The objective of this step is to evaluate the predictive performance of the model and derive optimism-corrected indices of discrimination (*C* index) and calibration (calibration slope and calibration-in-the-large).

To obtain estimates of optimism, we used bootstrap estimation with *J*=200 resamples. More details of this procedure can be found elsewhere (15). To illustrate the procedure, we describe the process for deriving the optimism-corrected *C* index but the process for the calibration slope follows a similar logic but using the coefficients of the linear predictors instead. Consider an imputed dataset *i*, we first regressed the survival outcome in imputed dataset *i* on the stable risk factors in imputed dataset *i*, and saved the coefficients. We then shrunk the coefficients by *S_i_* to obtain shrunken coefficients. We computed the LP by taking the linear combination of the predictors weighted by the shrunken coefficients. We regressed the outcome in imputed dataset *i* on the LP and obtained an apparent *C_i_* index.

Now consider a bootstrap resample *j* from the impute dataset *i*. We regressed the survival outcome in bootstrap dataset *j* on the stable risk factors in bootstrap dataset *j*, and saved the coefficients. We then shrunk the coefficients by *S_i_* to obtain shrunken coefficients. We then computed two linear predictors: LP*_boot_*, which is linear combination of the stable risk factors in the bootstrap sample *j* weighted by the shrunken coefficients; and LP*_test_* which is linear combination of the stable risk factors in the imputed dataset *i* weighted by the shrunken coefficients. We regressed the survival outcome in bootstrap sample *j* on LP*_boot_* and obtained *C_boot_*. We regressed the survival outcome in imputed dataset *i* on LP*_test_* and obtained *C_test_*. We then subtracted *C_boot_* from *C_test_* to get an index of optimism*_j_*. We averaged all optimism*_j_* across the *J* resamples to get a stable measure of optimism*_i_*. An optimism-corrected C for imputation *i* was then optimism-corrected *C_i_* = apparent *C_i_* minus optimism*_i_*. We then averaged all optimism-corrected *C_i_* across *I*=10 imputations using Rubin’s rules to obtain a single optimism-corrected *C* index (16).

| **Imputation** | **Optimism corrected C-index [95% CI]** | **Optimism-corrected calibration slope [95% CI]** |
| --- | --- | --- |
| 1 | 0.711 [0.649, 0.774] | 1.005 [0.530, 1.479] |
| 2 | 0.701 [0.641, 0.761] | 1.021 [0.516, 1.526] |
| 3 | 0.708 [0.648, 0.767] | 1.010 [0.498, 1.522] |
| 4 | 0.710 [0.647, 0.773] | 1.000 [0.503, 1.497] |
| 5 | 0.704 [0.635, 0.774] | 0.944 [0.386, 1.501] |
| 6 | 0.715 [0.658, 0.771] | 1.014 [0.537, 1.491] |
| 7 | 0.707 [0.646, 0.768] | 1.019 [0.564, 1.473] |
| 8 | 0.714 [0.655, 0.773] | 1.003 [0.490, 1.515] |
| 9 | 0.707 [0.650, 0.765] | 0.998 [0.568, 1.428] |
| 10 | 0.708 [0.643, 0.773] | 0.991 [0.397, 1.585] |
| **Average** | **0.709 [0.647, 0.771]** | **1.000 [0.495, 1.506]** |

For the 95% confidence intervals (CI) for each interval, the bootstrap standard error is calculated as the standard deviation of the empirical distribution of bootstrap estimates. For the 95% CI for the average, we pooled the within-imputation standard errors using Rubin’s rules to obtain a pooled standard error (17). Confidence intervals are calculated using the normal approximation.

**5. Presenting the predicted probability of RA flare.** The final equation of the prediction model is obtained by first estimating the coefficients of the stable risk factors from a Cox model in each imputation *i,* performing shrinkage using *S_i_*, and then pooling them using Rubin’s rules.

We then appended the rows of all imputed datasets *i* to create a stacked dataset (15). Because each participant has *I* replications in this stacked dataset, we gave each observation a weight of 1/*I*. We then computed the *prognostic index* (PI) as the linear combination of the values of the stable risk factors in the stacked dataset, weighted by the coefficients in the final equation of the prediction model. We regressed the survival outcome in the stacked dataset on the PI in a weighted Cox PH model, and obtained the value of the baseline survival function $\hat{S}_{0}\left( t \right)$ (valued at PI equals zero) at 30, 60, 90, 120, and 168 days post-DMARD cessation (see below). The weighted stacked dataset was also used in the computation of calibration plots.

| **Days after cessation of DMARD** | **Baseline survival function** |
| --- | --- |
| 30 | 0.969 |
| 60 | 0.876 |
| 90 | 0.801 |
| 120 | 0.714 |
| 168 | 0.672 |

| Procedures | Screening Visit | Day 0: Baseline (a)  Telephone consultation | Day 0: Baseline (b)  Synovial biopsy (OPTIONAL) | Day 14 | Day 35 | Day 56 | Day 84 | Day 168 | Patient-requested ad-hoc study visits | Visit 2 weeks following ad-hoc study visit | Synovial biopsy assessment visit after flare confirmed |
| --- | --- | --- | --- | --- | --- | --- | --- | --- | --- | --- | --- |
| Discuss Study / confirm willingness to continue participation in study | X | X | X | X | X | X | X | X | X | X | X |
| Informed Consent for study | X |  |  |  |  |  |  |  |  |  |  |
| Collect Demographics and medical history | X |  |  |  |  |  |  |  |  |  |  |
| Record Current medication | X |  |  | X | X | X | X | X | X | X |  |
| General Physical examination^[[1]](#footnote-2)^ | X |  |  |  |  |  |  |  |  |  |  |
| Rheumatological Assessment - DAS28-CRP | X |  |  | X | X | X | X | X | X | X |  |
| Instruction to discontinue DMARDs  (if not opting for synovial Biopsy) |  | X |  |  |  |  |  |  |  |  |  |
| Instruction to discontinue DMARDs (if opting for synovial biopsy) |  |  | X |  |  |  |  |  |  |  |  |
| Patient Reported Outcome Measures / Questionnaires | | | | | | | | | | | |
| HAQ-DI | X |  |  |  |  |  |  | X | X | X |  |
| RAPID-3 | X |  |  | X | X | X | X | X | X | X |  |
| EuroQol 5D-5L | X |  |  | X | X | X | X | X | X | X |  |
| MFI | X |  |  |  |  |  |  | X | X | X |  |
| RA-FQ | X |  |  | X | X | X | X | X | X | X |  |
| FLARE-RA | X |  |  | X | X | X | X | X | X | X |  |
| Blood tests | | | | | | | | | | | |
| Full Blood Count (FBC) | X |  |  | X | X | X | X | X | X | X |  |
| Inflammatory markers (ESR & CRP) | X |  |  | X | X | X | X | X | X | X |  |
| Antibodies (RF & ACPA) | X |  |  |  |  |  |  |  |  |  |  |
| Other clinical bloods (UE, LFT & Clotting) | X |  |  |  |  |  |  |  | X |  |  |
| Research blood tests (Serum, EDTA, Tempus and Heparinised samples) | X |  |  | X | X | X | X | X | X | X |  |
| Other research tests | | | | | | | | | | | |
| Urine Sample | X |  |  | X | X | X | X | X | X | X |  |
| Pregnancy test^[[2]](#footnote-3)^ | X |  |  |  |  |  |  |  |  |  |  |
| Stool Sample (OPTIONAL) | X |  |  | X | X | X | X | X | X | X |  |
| Ultrasound assessment for Synovial Biopsy (OPTIONAL AT BASELINE – additional consent required) |  |  | [X] |  |  |  |  |  |  |  | X |
| Accelerometer provided^[[3]](#footnote-4)^ (OPTIONAL) | X |  |  |  |  |  |  |  |  |  |  |
| Activity diary provided (OPTIONAL) | X |  |  | X | X | X | X |  | X |  |  |

## Supplementary Table 1: Schedule of events in the BIO-FLARE study.

**Adverse events**

In total, 82 out of 121 participants (68%) experienced at least one adverse event (AE) in the sample. There were a total of 155 adverse events with a median of 1 (IQR: 1, 2) event per participant (range: 1 to 6). The breakdown of the number of participants reporting each type of AE is presented below, organised by their system organ class. Additionally, there were 4 serious adverse events (SAE) occurring over 4 participants (Supplementary Table 3).

|  |  | Modified per-protocol cohort (n=111)* | |
| --- | --- | --- | --- |
|  | Study population (n=121) | Flared (n=58) | Remission at week 24 visit (n=53) |
|  | N (%) | N (%) | N (%) |
| **Blood and lymphatic system disorders** |  |  |  |
| Anaemia | 1 (0.8) | 1 (1.7) | 0 |
| Neutropenia | 2 (1.7) | 0 | 2 (3.8) |
| Thrombocytopenia | 1 (0.8) | 0 | 1 (1.9) |
| **Ear and labyrinth disorders** |  |  |  |
| Excessive cerumen production | 1 (0.8) | 0 | 1 (1.9) |
| Vertigo | 1 (0.8) | 0 | 1 (1.9) |
| **Endocrine disorders** |  |  |  |
| Hypothyroidism | 1 (0.8) | 0 | 1 (1.9) |
| **Eye disorders** |  |  |  |
| Blepharitis | 1 (0.8) | 0 | 1 (1.9) |
| Cataract | 1 (0.8) | 0 | 1 (1.9) |
| Dry eye | 1 (0.8) | 1 (1.7) | 0 |
| **Gastrointestinal disorders** |  |  |  |
| Constipation | 1 (0.8) | 0 | 1 (1.9) |
| Dyspepsia | 1 (0.8) | 0 | 1 (1.9) |
| Enteritis | 1 (0.8) | 1 (1.7) | 0 |
| Gastritis | 1 (0.8) | 0 | 1 (1.9) |
| Mouth ulceration | 1 (0.8) | 1 (1.7) | 0 |
| Pancreatic mass | 1 (0.8) | 0 | 1 (1.9) |
| Toothache | 2 (1.7) | 1 (1.7) | 1 (1.9) |
| Vomiting | 1 (0.8) | 0 | 1 (1.9) |
| **General disorders and administration site conditions** |  |  |  |
| Chest pain | 1 (0.8) | 0 | 1 (1.9) |
| Critical illness | 1 (0.8) | 1 (1.7) | 0 |
| Fatigue | 2 (1.7) | 2 (3.4) | 0 |
| Hernia | 1 (0.8) | 0 | 1 (1.9) |
| Malaise | 1 (0.8) | 0 | 1 (1.9) |
| **Immune system disorders** |  |  |  |
| Hypersensitivity | 1 (0.8) | 0 | 1 (1.9) |
| **Infections and infestations** |  |  |  |
| Cellulitis | 1 (0.8) | 1 (1.7) | 0 |
| Conjunctivitis viral | 1 (0.8) | 1 (1.7) | 0 |
| Coxsackie viral infection | 1 (0.8) | 0 | 1 (1.9) |
| Gastroenteritis | 1 (0.8) | 0 | 1 (1.9) |
| Gastroenteritis viral | 1 (0.8) | 0 | 1 (1.9) |
| Infected bite | 1 (0.8) | 1 (1.7) | 0 |
| Lower respiratory tract infection | 5 (4.1) | 1 (1.7) | 4 (7.5) |
| Oral herpes | 1 (0.8) | 0 | 0 |
| Otitis externa | 1 (0.8) | 1 (1.7) | 1 (1.9) |
| Rash pustular | 1 (0.8) | 1 (1.7) | 0 |
| Rhinitis | 1 (0.8) | 0 | 1 (1.9) |
| Sinusitis | 1 (0.8) | 1 (1.7) | 0 |
| Tooth abscess | 1 (0.8) | 0 | 1 (1.9) |
| Tooth infection | 1 (0.8) | 0 | 1 (1.9) |
| Upper respiratory tract infection | 20 (16.5) | 9 (15.5) | 8 (15.1) |
| Urinary tract infection | 1 (0.8) | 0 | 0 |
| Viral infection | 1 (0.8) | 1 (1.7) | 0 |
| Viral upper respiratory tract infection | 7 (5.8) | 6 (10.3) | 1 (1.9) |
| **Injury, poisoning and procedural complications** |  |  |  |
| Arthropod bite | 1 (0.8) | 0 | 1 (1.9) |
| Avulsion fracture | 1 (0.8) | 0 | 1 (1.9) |
| Back injury | 1 (0.8) | 0 | 1 (1.9) |
| Contusion | 1 (0.8) | 1 (1.7) | 0 |
| Fall | 2 (1.7) | 1 (1.7) | 1 (1.9) |
| Laceration | 1 (0.8) | 1 (1.7) | 0 |
| Limb injury | 1 (0.8) | 1 (1.7) | 0 |
| Spinal fracture | 2 (1.7) | 2 (3.4) | 0 |
| Wound | 2 (1.7) | 1 (1.7) | 1 (1.9) |
| **Investigations** |  |  |  |
| Blood glucose abnormal | 1 (0.8) | 1 (1.7) | 0 |
| C-reactive protein increased | 1 (0.8) | 1 (1.7) | 0 |
| Liver function test abnormal | 1 (0.8) | 1 (1.7) | 0 |
| Platelet count decreased | 1 (0.8) | 1 (1.7) | 0 |
| **Musculoskeletal and connective tissue disorders** |  |  |  |
| Arthralgia | 1 (0.8) | 0 | 1 (1.9) |
| Fracture | 1 (0.8) | 0 | 1 (1.9) |
| Joint stiffness | 1 (0.8) | 1 (1.7) | 0 |
| Musculoskeletal pain | 1 (0.8) | 1 (1.7) | 0 |
| Myalgia | 1 (0.8) | 1 (1.7) | 0 |
| Pain in extremity | 1 (0.8) | 1 (1.7) | 0 |
| Periarthritis | 1 (0.8) | 1 (1.7) | 0 |
| Soft tissue swelling | 1 (0.8) | 0 | 1 (1.9) |
| Tendonitis | 2 (1.7) | 1 (1.7) | 1 (1.9) |
| Tenosynovitis | 2 (1.7) | 2 (3.4) | 0 |
| **Neoplasms benign, malignant and unspecified (incl cysts and polyps)** |  |  |  |
| Seborrhoeic keratosis | 1 (0.8) | 0 | 0 |
| **Nervous system disorders** |  |  |  |
| Cerebrovascular accident | 1 (0.8) | 0 | 0 |
| Dizziness | 1 (0.8) | 1 (1.7) | 0 |
| Headache | 3 (2.5) | 1 (1.7) | 2 (3.8) |
| Migraine | 1 (0.8) | 0 | 1 (1.9) |
| Neuralgia | 1 (0.8) | 1 (1.7) | 0 |
| Restless legs syndrome | 1 (0.8) | 0 | 1 (1.9) |
| Sciatica | 4 (3.3) | 2 (3.4) | 2 (3.8) |
| Seizure | 1 (0.8) | 0 | 1 (1.9) |
| Syncope | 1 (0.8) | 0 | 1 (1.9) |
| Transient ischaemic attack | 1 (0.8) | 0 | 0 |
| **Psychiatric disorders** |  |  |  |
| Depressed mood | 1 (0.8) | 0 | 1 (1.9) |
| Emotional distress | 1 (0.8) | 1 (1.7) | 0 |
| Insomnia | 1 (0.8) | 1 (1.7) | 0 |
| **Respiratory, thoracic and mediastinal disorders** |  |  |  |
| Cough | 5 (4.1) | 2 (3.4) | 3 (5.7) |
| Nasal dryness | 1 (0.8) | 1 (1.7) | 0 |
| Oropharyngeal pain | 6 (5) | 4 (6.9) | 2 (3.8) |
| **Skin and subcutaneous tissue disorders** |  |  |  |
| Eczema | 1 (0.8) | 0 | 1 (1.9) |
| Neurodermatitis | 1 (0.8) | 0 | 1 (1.9) |
| Pruritus | 1 (0.8) | 1 (1.7) | 0 |
| Rash | 1 (0.8) | 1 (1.7) | 1 (1.9) |
| Rash erythematous | 2 (1.7) | 1 (1.7) | 0 |
| Skin lesion | 1 (0.8) | 1 (1.7) | 0 |
| Transient acantholytic dermatosis | 1 (0.8) | 1 (1.7) | 0 |
| **Surgical and medical procedures** |  |  |  |
| Medical device removal | 1 (0.8) | 0 | 1 (1.9) |
| Tooth extraction | 2 (1.7) | 1 (1.7) | 1 (1.9) |
| Tooth repair | 1 (0.8) | 1 (1.7) | 0 |
| **Vascular disorders** |  |  |  |
| Aneurysm | 1 (0.8) | 0 | 1 (1.9) |
| Hypertension | 1 (0.8) | 1 (1.7) | 0 |
| Temporal arteritis | 1 (0.8) | 0 | 0 |

## Supplementary Table 2: All adverse events. *Discrepancy between study population versus modified per-protocol cohort is due to exclusion of participants who were lost to follow-up (n=7) or withdrawn (n=3) before week 24 visit

| **Participant** | **Days from DMARD cessation to start of SAE** | **Duration of SAE (days)** | **SAE** | **Causality** | **Expected** | **Severity** | **Type of SAE / Action taken** | **Patient withdrawn from study?** |
| --- | --- | --- | --- | --- | --- | --- | --- | --- |
| 1 | 174 |  | Giant cell arteritis | Unrelated |  | Mild | Hospitalisation | Yes |
| 2 | 176 | 2 | Headache | Unrelated |  | Moderate | Hospitalisation | No |
| 3 | 92 | n/a | Incidental pancreatic body cystic mass | Unrelated | Unexpected | Severe | Other medically significant event – referred for urgent investigation | No |
| 4 | 99 | 1 | Brief hospital admission for atypical chest pain | Unrelated | Unexpected | Moderate | Hospitalisation | No |

## Supplementary Table 3: Serious adverse events

| Patient | Description |
| --- | --- |
| 1 | 1 tender joint (right wrist), patient VAS = 51/100, CRP = 8 giving a DAS28-CRP of 3.03. Clinical team and patient felt they were flaring so a shared decision was made to restart DMARDs rather than wait for a second ad-hoc appointment in 14 days to confirm flare |
| 2 | Ankle (tibialis posterior) tenosynovitis requiring treatment. |
| 3 | Bilateral knee synovitis; three swollen joints in total; clinicians and patient felt restarting DMARDs necessary. |

## Supplementary Table 4: Characteristics of patients adjudged to have flared based on clinician discretion (n=3).

|  | Imputation | | | | | | | | | | Average BIF |
| --- | --- | --- | --- | --- | --- | --- | --- | --- | --- | --- | --- |
|  | 1 | 2 | 3 | 4 | 5 | 6 | 7 | 8 | 9 | 10 |  |
| Age | 0.105 | 0.115 | 0.105 | 0.110 | 0.105 | 0.110 | 0.120 | 0.135 | 0.130 | 0.110 | 0.115 |
| **Sex** | 0.585 | 0.605 | 0.590 | 0.585 | 0.585 | 0.595 | 0.605 | 0.525 | 0.555 | 0.595 | 0.583 |
| Time from diagnosis to baseline | 0.170 | 0.175 | 0.155 | 0.215 | 0.205 | 0.240 | 0.205 | 0.150 | 0.200 | 0.155 | 0.187 |
| Time from symptom onset to DMARD commencement | 0.425 | 0.345 | 0.370 | 0.350 | 0.335 | 0.345 | 0.375 | 0.350 | 0.340 | 0.240 | 0.348 |
| **Methotrexate use** | 0.775 | 0.790 | 0.795 | 0.780 | 0.765 | 0.765 | 0.770 | 0.750 | 0.740 | 0.750 | 0.768 |
| **RF** | 0.865 | 0.885 | 0.870 | 0.865 | 0.890 | 0.900 | 0.840 | 0.915 | 0.925 | 0.920 | 0.888 |
| **ACPA** | 0.730 | 0.775 | 0.710 | 0.835 | 0.720 | 0.590 | 0.785 | 0.605 | 0.460 | 0.560 | 0.677 |
| DAS28-CRP | 0.155 | 0.150 | 0.155 | 0.155 | 0.150 | 0.160 | 0.155 | 0.140 | 0.160 | 0.145 | 0.153 |
| ACR/EULAR Boolean Remission | 0.200 | 0.185 | 0.185 | 0.195 | 0.175 | 0.165 | 0.180 | 0.175 | 0.165 | 0.190 | 0.182 |
| Education | 0.215 | 0.200 | 0.225 | 0.205 | 0.220 | 0.250 | 0.285 | 0.260 | 0.230 | 0.275 | 0.237 |
| BMI | 0.380 | 0.305 | 0.445 | 0.380 | 0.520 | 0.485 | 0.545 | 0.370 | 0.535 | 0.485 | 0.445 |
| Current smoking | 0.190 | 0.195 | 0.220 | 0.205 | 0.245 | 0.235 | 0.250 | 0.175 | 0.220 | 0.225 | 0.216 |
| Any alcohol use | 0.465 | 0.435 | 0.455 | 0.430 | 0.435 | 0.465 | 0.430 | 0.420 | 0.445 | 0.445 | 0.443 |
| CCI | 0.195 | 0.195 | 0.200 | 0.195 | 0.175 | 0.200 | 0.190 | 0.190 | 0.185 | 0.190 | 0.192 |
| Corticosteroid use | 0.260 | 0.240 | 0.235 | 0.240 | 0.240 | 0.255 | 0.245 | 0.235 | 0.260 | 0.255 | 0.247 |

## Supplementary Table 5: Bootstrap inclusion frequencies

Sex, methotrexate use, RF, and ACPA were brought forward to the prediction model. Although sex did not cross the a priori 60% average BIF threshold, we included it in the prediction model as its average BIF was highly proximal to the threshold. RF=Rheumatoid Factor; ACPA=Anti-Citrullinated Peptide Antibody; BMI=Body Mass Index; CCI=Charlson Comorbidity Index. Bolded predictors are those that were included in the prediction model


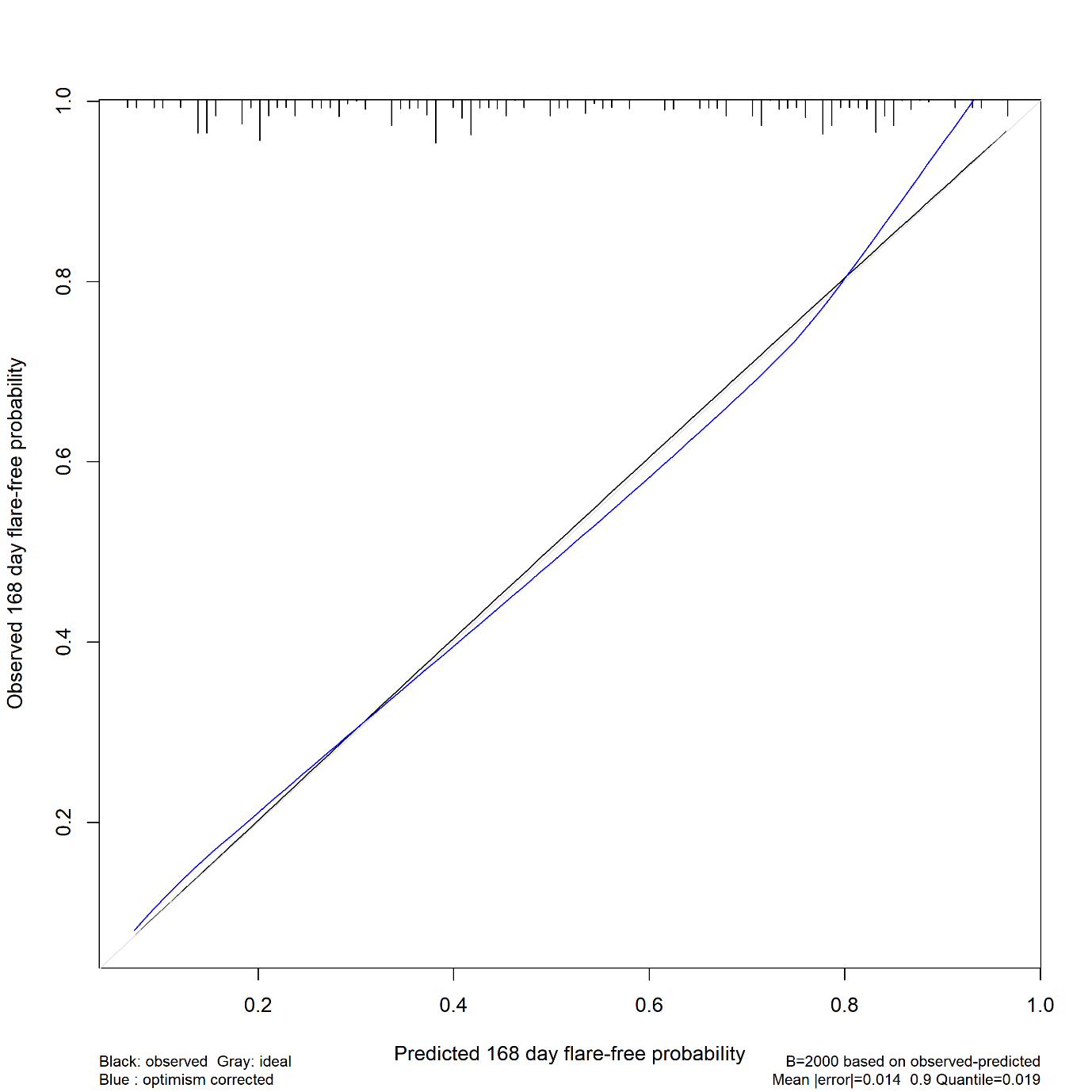


## Supplementary Figure 1: Calibration plot at day 168

Dashed lines at the top represent a histogram of the predicted 168-day flare-free probabilities. The risk (or probability) of flare by 168 days may be taken as 1.0 minus the flare-free probability. The light grey diagonal line represents the line of perfect agreement between predicted and observed flare-free probabilities. The blue line indicates the optimism-corrected calibration curve, and the black line indicates the uncorrected calibration curve. The results suggest that the model produced predicted estimates of flare risk that had good agreement with observed risk. As a minor caveat, based on the calibration slopes for risk of flare by 168 days, the model slightly underestimated the predicted risk of flare for participants with an observed “moderate-high” risk (30–80% observed risk), and overestimated predicted risk of flare for participants at lower (≤30% observed risk) and higher observed risk (≥80% observed risk).


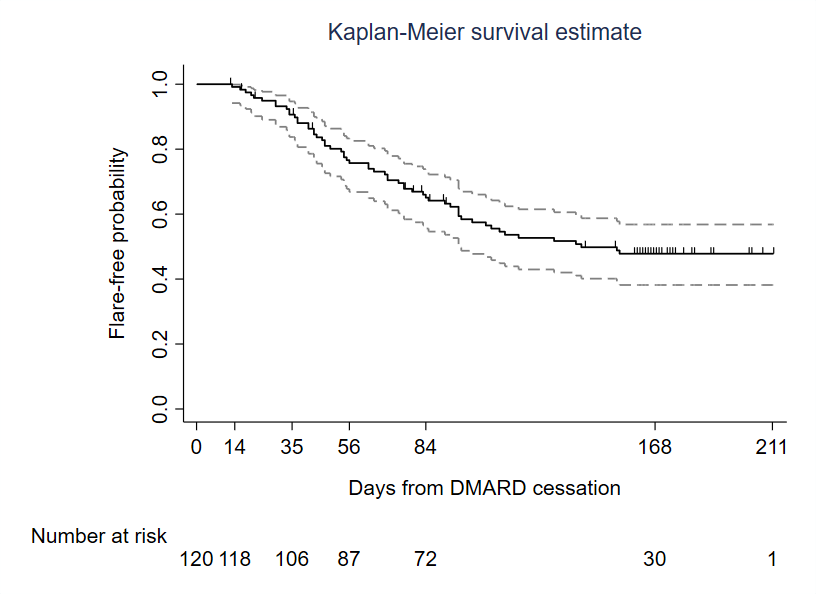


## Supplementary Figure 2: Kaplan-Meier plot of flare-free survival based on face-to-face visits.

Solid line is the Kaplan-Meier estimate of the survival function, the grey dashed lines are the 95% CI, and vertical black marks indicate censoring. Outcomes defined as per sensitivity analysis, i.e. using last face-to-face visits / last available DAS28-CRP.

## Supplementary References

1. Riley RD, Snell KI, Ensor J, Burke DL, Harrell Jr FE, Moons KG, et al. Minimum sample size for developing a multivariable prediction model: PART II - binary and time-to-event outcomes. Statistics in Medicine. 2019;38(7):1276-96.

2. Riley RD. Correction to: Minimum sample size for developing a multivariable prediction model: Part II‐binary and time‐to‐event outcomes by Riley RD, Snell KI, Ensor J, et al. Statistics in Medicine. 2019;38(30):5672-.

3. Baker KF, Skelton AJ, Lendrem DW, Scadeng A, Thompson B, Pratt AG, et al. Predicting drug-free remission in rheumatoid arthritis: A prospective interventional cohort study. Journal of autoimmunity. 2019;105:102298.

4. Bykerk VP, Massarotti EM. The new ACR/EULAR remission criteria: rationale for developing new criteria for remission. Rheumatology. 2012;51(suppl 6):vi16-vi20.

5. Charlson ME, Pompei P, Ales KL, MacKenzie CR. A new method of classifying prognostic comorbidity in longitudinal studies: development and validation. Journal of chronic diseases. 1987;40(5):373-83.

6. van Buuren SG-O, K. mice: Multivariate Imputation by Chained Equations in R. HJournal of Statistical Software. 2011;45(3):1-67.

7. Marshall A, Altman DG, Holder RL. Comparison of imputation methods for handling missing covariate data when fitting a Cox proportional hazards model: a resampling study. BMC Medical Research Methodology. 2010;10(1):112.

8. White IR, Royston P. Imputing missing covariate values for the Cox model. Statistics in Medicine. 2009;28(15):1982-98.

9. Heymans MW, Van Buuren S, Knol DL, Van Mechelen W, De Vet HC. Variable selection under multiple imputation using the bootstrap in a prognostic study. BMC Medical Research Methodology. 2007;7(1):33.

10. Sauerbrei W, Buchholz A, Boulesteix A-L, Binder H. On stability issues in deriving multivariable regression models. Biometrical Journal. 2015;57(4):531-55.

11. Sauerbrei W, Schumacher M. A bootstrap resampling procedure for model building: Application to the cox regression model. Statistics in medicine. 1992;11(16):2093-109.

12. Altman DG, Andersen PK. Bootstrap investigation of the stability of a cox regression model. Statistics in medicine. 1989;8(7):771-83.

13. Sauerbrei WM-H, C; Benner, A; Royston, P. Multivariable regression model building by using fractional polynomials: Description of SAS, STATA and R programs. Computational Statistics & Data Analysis. 2006;50:3464 - 85.

14. Riley RD, Snell KIE, Martin GP, Whittle R, Archer L, Sperrin M, et al. Penalization and shrinkage methods produced unreliable clinical prediction models especially when sample size was small. Journal of Clinical Epidemiology. 2021;132:88-96.

15. Steyerberg EW. Clinical Prediction Models: A Practical Approach to Development, Validation, and Updating: Springer Cham; 2019. XXXIII, 558 p.

16. Marshall A, Altman DG, Royston P, Holder RL. Comparison of techniques for handling missing covariate data within prognostic modelling studies: a simulation study. BMC Medical Research Methodology. 2010;10(1):7.

17. Bartlett JW, Hughes RA. Bootstrap inference for multiple imputation under uncongeniality and misspecification. Stat Methods Med Res. 2020 Dec;29(12):3533-3546.

1. Depending on the circumstances of the consultation, physical examination may be indicated at any study visit to establish whether DAS28-CRP reflects arthritis activity or infection etc. General Physical Examination is only mandatory at Screening. [↑](#footnote-ref-2)
2. Mandatory at Screening but should be performed at any visit subsequently if routine questioning suggests a participant may be pregnant. Serum or urine tests to be performed subsequently in line with local policy [↑](#footnote-ref-3)
3. This may be provided after the study visit once eligibility confirmed, either by post, or at the optional Baseline Synovial Biopsy Visit (if applicable) [↑](#footnote-ref-4)
