# Supplementary material for: Clinical predictors of flare and drug-free remission in rheumatoid arthritis: preliminary results from the prospective BIO-FLARE experimental medicine study
Source: BMJ Open. 2025 Apr 9;15(4):e092478. doi: 10.1136/bmjopen-2024-092478 (PMC11987156; doi:10.1136/bmjopen-2024-092478)
Supplement: Supplementary file 2 [file bmjopen-2024-092478.reviewer_comments.pdf]

PEER REVIEW HISTORY

BMJ Open publishes all reviews undertaken for accepted manuscripts. Reviewers are asked to complete a checklist review form and are provided with free text boxes to elaborate on their assessment. These free text comments are reproduced below.

ARTICLE DETAILS

Title (Provisional)

Clinical predictors of flare and drug-free remission in rheumatoid arthritis: preliminary results from the prospective BIO-FLARE experimental medicine study

Authors

Rayner, Fiona; Hiu, Shaun; Melville, Andrew; Bigirumurame, Theophile; Anderson, Amy; Dyke, Bernard; Kerrigan, Sean; McGucken, Andrew; Prichard, Jonathan; Shahrokhabadi, Mohadeseh Shojaei; Hilkens, Catharien M U; Buckley, Christopher D; McInnes, Iain B; Ng, Wan-Fai; Goodyear, Carl; Teare, Dawn; Filer, Andrew; Siebert, Stefan; Raza, Karim; Pratt, Arthur; Baker, Kenneth F; Isaacs, John

---

VERSION 1 - REVIEW

**Reviewer**	**1**
**Name**	**Richter, Adrian**
**Affiliation**	**German Rheumatism Research Center Berlin, Epidemiology and Health Services Research**
**Date**	**07-Oct-2024**
**COI**	**None**

Review of the manuscript entitled: „Clinical predictors of flare and drug-free remission in rheumatoid arthritis: preliminary results from the prospective BIO-FLARE experimental medicine study".

The authors developed a model to predict disease flares in rheumatoid arthritis after discontinuation of DMARD therapy. The methods used to train and test the model appear plausible, although some inconsistencies and uncertainties in modeling decisions are apparent.

Major

(1) Please state clearly in the objectives that this study aimed at developing a prediction model.

(2) Please mention in the Strength and Limitations section, that this model has been developed using a very small number of individuals.

(3) Is it possible to examine how representative the population is?

(4) For this type of study it is recommended to adhere to reporting guidelines for the development of prediction models and to present this information in the supplement (Collins et al., 2015).

(5) Please state why non-linear forms of predictors are restricted to RF and ACPA. Age, disease duration, DAS28, and further may also be associated in non-linear form with the outcome.

(6) Please elaborate on the decision for the final functional form of ACPA. This is not clear from the supplement.

(7) Prior calculation of the maximum number of variables included in the prediction model is not comprehensible. Why is this number not a result of the model training and validation process?

(8) Please specify the “problems with convergence for the employment variable”.

(9) Using 200 bootstrap samples appears quite small or is the process repeated across all imputations?

(10) The Kaplan-Meier plot in the supplements shows considerable amount of censoring. Please explain what drives this censoring process, did patients drop out of the study? Did the authors investigate reasons for dropout and characteristics of patients dropping out?

Minor

(1) Could the disease activity be recovered in those who experienced flares or is lasting damage to be expected?

(2) This reviewer misses a conclusion as to whether discontinuation of DMARD therapy is recommended at all.

Collins, G. S., Reitsma, J. B., Altman, D. G., & Moons, K. G. M. (2015). Transparent reporting of a multivariable prediction model for individual prognosis or diagnosis (TRIPOD): the TRIPOD Statement. BMC medicine, 13(1), 1.
https://doi.org/https://doi.org/10.1186/s12916-014-0241-z

**Reviewer**	**2**
**Name**	**Bozzalla-Cassione, Emanuele**
**Affiliation**	**University of Pavia, Rheumatology**
**Date**	**08-Jan-2025**
**COI**	**None**

The manuscript presents a well-designed study model that seeks to enhance our understanding of flare occurrence following csDMARD withdrawal and to predict the

likelihood of achieving drug-free remission. The results are articulated, tough they remain preliminary, as acknowledged by the authors, and do not yet provide substantial new insights into flare prediction in the context of drug-free remission. Nevertheless, the effort to develop an algorithm to support clinical decision-making is a compelling and innovative approach that adds value to the manuscript.

1. In the introduction, you state that DMARD cessation is “endorsed by international treatment guidelines.” Please provide appropriate references to support this statement.

2. Were the included RA patients treated according to a treat-to-target approach? If so, could this have influenced the outcomes?

3. In the inclusion criteria (n°2), it is stated that patients were required to maintain a stable csDMARD dose, with no dose increase in the six months prior to the screening visit. Does this also imply that no dose tapering was allowed in the same period? If so, please clarify this explicitly.

4. The flare criteria applied in the current study appear complex. While the inclusion of flare reasons in the results section is highly appreciated, the decision to merge a strict clinimetric outcome with a more subjective, medical-based outcome may be confusing for readers. Could you elaborate on and justify this choice?

5. Patients were not required to achieve stable or persistent remission prior to treatment cessation. This is a notable choice, as:

1) It diverges from many drug-free remission study protocols, where persistent remission is typically required, and it could be anticipated that this approach may result in a higher flare rate.

2) It significantly diverges from the current and past (since at least 2016) EULAR recommendations for tapering, which emphasize persistent remission as an entry criterion for dose reduction.

Could you discuss the rationale behind this approach and its potential implications?

6. The methods section does not specify the time interval between the screening visit and baseline. Additionally, at baseline, no face-to-face visits are scheduled, and no clinical data are collected (as noted in Supplementary Table 1). Given that disease status is dynamic, this interval may be relevant. Please include this information.

7. Among the 16 variables included in the analysis based on subject knowledge, methotrexate (MTX) use at the baseline visit was included, but the MTX dose (which varies across your cohort) was not. While the decision to include a variable applicable across the entire cohort is understandable, MTX dose could hold significant relevance in a protocol where abrupt cessation without tapering is applied, especially given that most patients were on MTX (mono- or combination therapy) at baseline. Could you provide additional sub-analyses including MTX dose as a variable?

8. Regarding Figure 1:

1) In the orange square, please correct the range (2.4–3.2); 3.2 is already considered a flare.

2) The "medical-based flare" criterion (n°3 described in the methods section) is missing from Figure 1. Please include it.

9. In the supplementary material, under the "Adverse Events" section, you refer to Supplementary Tables 5 and 6. This appears to be a typo—please correct.

10. Minor: Please rearrange the order of the supplementary material so that all tables and figures appear consecutively. This would improve readability.

VERSION 1 - AUTHOR RESPONSE

	Comments	Response
Reviewer 1 Major		
1.	Please state clearly in the objectives that this study aimed at developing a prediction model.	This has been added as an objective in the abstract, objectives section
2.	Please mention in the Strength and Limitations section, that this model has been developed using a very small number of individuals.	A new bullet point has been added in strengths and limitations
3.	Is it possible to examine how representative the population is?	There is no reason to believe that that study population are significantly different to another population of patients with rheumatoid arthritis in remission who wish to stop their DMARDs. The study population were predominantly women (reflecting the female predominance of rheumatoid arthritis) and white (reflecting the geographical location of the recruiting hospitals). There was a predominance of retired people in the study, likely reflecting on the ability of attending more frequent research study visits than those in employment.
4.	For this type of study it is recommended to adhere to reporting guidelines for the development of prediction models and to present this information in the supplement (Collins et al., 2015).	Thank you to the reviewer for highlighting this and the associated paper. We have included the TRIPOD checklist which has been uploaded as a supplementary material.
5.	Please state why non-linear forms of predictors are	Thank you to the reviewer for raising this. Yes, we only explored non-linear functional forms

	restricted to RF and ACPA. Age, disease duration, DAS28, and further may also be associated in non-linear form with the outcome.	following the variable selection strategy. This was due to the challenge posed by grouping terms for the same explanatory variable to ensure they 'stayed together' or were 'dropped together' in an elastic net. Hence, to simplify the process, variable selection was run with linear terms only, then we applied non-linear transformations after the selection process.<br>We have added this in "2. Assessment of non-linear forms for continuous covariates" section of the supplement to highlight this limitation in our approach.
6.	Please elaborate on the decision for the final functional form of ACPA. This is not clear from the supplement.	Thank you for raising this oversight. We have clarified in "2. Assessment of non-linear forms for continuous covariates" section of the supplement, that we based our decision on the transformation that was suggested most frequently over the imputations.
7.	Prior calculation of the maximum number of variables included in the prediction model is not comprehensible. Why is this number not a result of the model training and validation process?	Apologies. The calculation presented in the supplement was a heavily simplified version of what was originally in a statistical analysis plan. There was a concern that the clinical readership may have found the original version too technical. We have now reproduced what was in the SAP in a new subsection "0. Justification of maximum number of predictors in model" and have included it as a supplement.<br>We used the methodology of Riley et al (references 1 and 2 of supplement) as it allowed us to use information from a previous prediction model of rheumatoid arthritis flare using biomarkers; the study had slightly different population and a different study design. This formula-driven approach is supported by Steyerberg (2019) chapter 3.7 (reference 15 of supplement).<br>Data-driven methods like the one the reviewer is suggesting is also recommended by Steyerberg (2019) but there is no evidence that one is superior over the other. We favoured the formula-driven approach as we had prior information that we were able to capitalise on.
8.	Please specify the "problems with convergence for the employment variable".	We have now clarified this. The convergence problems were due to there being a very low frequency in the unemployed subgroup. We have amended the text under Table 2.
9.	Using 200 bootstrap samples appears quite small or is the process repeated across all imputations?	Thanks for raising this. It is repeated across all imputations. The value of B=200 was used to expedite computation time. Given that our results for the estimation of the shrinkage factor, C index, and calibration slope (which used

		bootstrapping within an imputed dataset) are quite similar across the imputations, we do not feel that increasing the value of B would provide additional benefit.
10.	The Kaplan-Meier plot in the supplements shows considerable amount of censoring. Please explain what drives this censoring process, did patients drop out of the study? Did the authors investigate reasons for dropout and characteristics of patients dropping out?	Most of the censoring (vertical lines) on the KM plot are situated towards the 24-week visit mark, and that is because these individuals are being censored at their end of study visit. These visits allow for a +/- 7 day window and have not strictly been at 168-days.
Reviewer 1 minor		
1.	Could the disease activity be recovered in those who experienced flares or is lasting damage to be expected?	Previous published work investigating this has found that participants who flare regain remission quickly after restarting their usual medication. We do not have long term data from this study to corroborate this, although it would be an interesting future work to undertake. The above has been added to the discussion.
2.	This reviewer misses a conclusion as to whether discontinuation of DMARD therapy is recommended at all.	DMARD tapering and possible cessation is a topic that is often discussed when patients achieve remission or low disease activity. This is particularly the case if patients are experiencing side effects from medications. The aim of the study was not to ascertain whether DMARD cessation should be recommended or not, but to aid the decision making around DMARD cessation, for those patients and clinicians who want to consider it, and to allow a more informed decision based on the clinical factors that are present.
Reviewer 2 Major		
1.	In the introduction, you state that DMARD cessation is "endorsed by international treatment guidelines." Please provide appropriate references to support this statement.	Thank you for pointing this out, "endorsed by international treatment guidelines" has been removed. However, DMARD cessation is an acknowledged practice whose implementation presents a challenge to physicians and a knowledge gap that is widely acknowledged in the literature (e.g. https://pubmed.ncbi.nlm.nih.gov/27261493/).
2.	Were the included RA patients treated according to a treat-to-target approach? If so, could this	Rheumatoid arthritis patients enrolled into the BIO-FLARE study had been treated according to routine practice at participating recruitment sites, with a median disease duration of 6 years. In the majority of cases this will have comprised a treat-to-target approach. Since our intention

	have influenced the outcomes?	was to identify markers predictive of remission and flare amongst csDMARD-treated RA patients in remission under routine care, we consider the broad representativeness of our cohort to be appropriate and do not think this issue will have impacted outcomes unduly.
3.	In the inclusion criteria (n°2), it is stated that patients were required to maintain a stable csDMARD dose, with no dose increase in the six months prior to the screening visit. Does this also imply that no dose tapering was allowed in the same period? If so, please clarify this explicitly.	Dose reductions were permitted in this time period. It was stated that patients should be on a "stable dose" of DMARDs prior to enrollment, explicitly stating no increases in the last 6 months, but any dose reductions were left at the discretion of the PI as to whether this qualified as a stable dose.
4.	The flare criteria applied in the current study appear complex. While the inclusion of flare reasons in the results section is highly appreciated, the decision to merge a strict clinimetric outcome with a more subjective, medical-based outcome may be confusing for readers. Could you elaborate on and justify this choice?	We wanted to keep this study pragmatic in nature, to mirror the everyday decisions that are made about drug tapering and cessation in rheumatology clinics. Therefore it was important to add in a clinical discretion clause to the flare criteria. This helps to reassure ethical advisory boards, patients interested in the study and clinicians referring into the study that we tried to minimise the participant coming to any unnecessary harm. Flare based on clinical discretion was always discussed in detail with the PI overseeing the site, and often discussed in the wider TMG with the CI as well. Clinical discretion was only used in 3/58 cases (allowing for classification of flare if occurring in joints not included within the DAS28 assessment), although we agree that it does combine a subjective with an objective measure.
5.	Patients were not required to achieve stable or persistent remission prior to treatment cessation. This is a notable choice, as:<br>1) It diverges from many drug-free remission study protocols, where persistent remission is typically required, and it could be anticipated that this approach may result in a higher flare rate.	Thank you for raising this point.<br>1. Patients were recruited to this study from routine rheumatology clinics across numerous hospital sites. We therefore did not have quantitative data on disease activity prior to study enrolment. Whilst a "run-in" observational-only period for 12 months prior to DMARD cessation could have been included in the study protocol, this would have considerably lengthened the follow-up duration (extending from 6 to 18 months), with consequent burden of additional study visits for participants. It

	2) It significantly diverges from the current and past (since at least 2016) EULAR recommendations for tapering, which emphasize persistent remission as an entry criterion for dose reduction.<br>Could you discuss the rationale behind this approach and its potential implications?	was therefore agreed to incorporate a surrogate definition of sustained remission in the inclusion/exclusion criteria (i.e. no use of intra-articular or systemic glucocorticoids for 3 months prior to enrolment, and no escalation of DMARD therapy for 6 months prior to enrolment). Participants were all counselled regarding the risk of flare on DMARD cessation (specifically they were quoted the 50% flare risk) when consenting to the study, and were happy to proceed.<br>2. We have shown in our data that despite the fact that remission was only needed for a minimum of 6 months, this has not affect the flare rate, as the rate of roughly 50% flare corroborates previous published data.
6.	The methods section does not specify the time interval between the screening visit and baseline. Additionally, at baseline, no face-to-face visits are scheduled, and no clinical data are collected (as noted in Supplementary Table 1). Given that disease status is dynamic, this interval may be relevant. Please include this information.	Those participants that did not consent to a baseline synovial biopsy stopped their DMARDs immediately after eligibility had been determined, therefore they had no separate baseline visit. The eligibility and decision to stop DMARDs was communicated by a telephone call, usually the day after the screening visit, when blood tests were available. Those participants that consented to a synovial biopsy had this performed within 14 days of their screening visit. If there was any concern that their arthritis activity had changed between the screening visit and the synovial biopsy, clinical discretion could be used to re-assess the participant and decide regarding suitability for entering the study. The wording has been slightly amended in 2.2 Procedures and definitions to make this clearer.
7.	Among the 16 variables included in the analysis based on subject knowledge, methotrexate (MTX) use at the baseline visit was included, but the MTX dose (which varies across your cohort) was not. While the decision to include a variable applicable across the entire cohort is understandable, MTX dose	Thank you for the comment and we agree that methotrexate dose could hold relevance in this case. There were a number of reasons that methotrexate dose was not included in the variables, that we have explained below:<br>1. Medication dose variables are what are considered sparse data – as there'll be a spike at 0 (for people who aren't taking any), then non-zero counts at several discrete and non-contiguous points (e.g. 5, 10, 20, etc).<br>2. Modelling it directly as a continuous predictor is unlikely to be useful as there

	could hold significant relevance in a protocol where abrupt cessation without tapering is applied, especially given that most patients were on MTX (mono- or combination therapy) at baseline. Could you provide additional sub-analyses including MTX dose as a variable?	is a systematic discontinuity in the allowable values, hence it is more valid to treat it as a discrete variable.<br>3. Modelling it as a discrete (categorical) variable, by treating each dose level as a dummy variable, is more ideal but still undesirable as there will be very small numbers at higher doses.<br>4. There is also the issue described in our response to reviewer 1 point 5. We were not sure how to make it such that either all dose level variables stay in the model or get selected out of the model together.<br>5. So it is through the most pragmatic of choices to dichotomise as users vs non-users (or 0 vs >0 dose).
8.	Regarding Figure 1:<br><br>1) In the orange square, please correct the range (2.4–3.2); 3.2 is already considered a flare.<br>2) The "medical-based flare" criterion (n°3 described in the methods section) is missing from Figure 1. Please include it.	Amended as suggested, with footnote regarding medical-based flare
9.	In the supplementary material, under the "Adverse Events" section, you refer to Supplementary Tables 5 and 6. This appears to be a typo—please correct.	Thank you for pointing this out, it has now been amended
10.	Minor: Please rearrange the order of the supplementary material so that all tables and figures appear consecutively. This would improve readability.	Amended

VERSION 2 - REVIEW

**Reviewer**	**2**
**Name**	**Bozzalla-Cassione, Emanuele**

**Affiliation**	**University of Pavia, Rheumatology**
**Date**	**06-Mar-2025**
**COI**	

The authors thoroughly answered all my questions and improved the text in agreement. I have no further comments.
